# H_2_O‑Dissociative Adsorption on Mg(0001) Surface—Corrosion Process under Atmospheric Conditions

**DOI:** 10.1021/acsomega.6c00009

**Published:** 2026-05-01

**Authors:** Yunyan Han, Haijun Jiao

**Affiliations:** † Key Laboratory of Eco-functional Polymer Materials of the Ministry of Education, College of Chemistry and Chemical Engineering, 12435 Northwest Normal University, Lanzhou 730070, China; ‡ 28392 Leibniz-Institut für Katalyse e.V. (LIKAT), Albert-Einstein-Str. 29A, Rostock 18059, Germany

## Abstract

The high susceptibility to corrosion of metallic magnesium, the lightest structural metal with great application potential, limits its practical use, and the corrosion mechanism, especially under atmospheric conditions, remains unclear. In this work, reactions of H_2_O on the Mg(0001) surface under atmospheric conditions along with the influence of oxygen on the H_2_O–Mg­(0001) interaction were investigated by DFT calculation. Electronic structure analyses (PDOS, COHP, and Bader charge) were conducted to provide a fundamental atomic-level explanation for the adsorption and reaction. Results show that molecular H_2_O adsorption on pure Mg(0001) is weak, dissociative adsorption is favored kinetically and thermodynamically, and the formed H* and OH* prefer surface sites, while O* prefers subsurface sites from low to high coverage for deep oxidation. Surface oxygen O* atoms, readily formed from the dissociation of O_2_ or H_2_O atoms, significantly enhance water dissociative adsorption. The oxidized surface layers saturated with hydroxyl layers, which will hinder the further reaction of the compounds of O_2_ and H_2_O on the surface, are protective under certain conditions.

## Graphical abstract

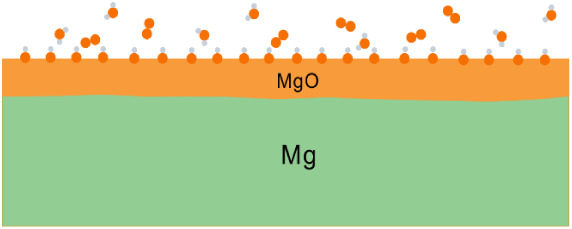

## 1 Introduction

Magnesium (Mg), the lightest structural metal and one of the most abundant metallic elements, has great application potential in transportation, aerospace, automotive, electronics, biomedical products, energy storage, and communications industries.^1–3^ Despite these potential advantages, Mg and its alloys have practical drawbacks such as high chemical activity and corrosivity due to the easy oxidation of the outer surfaces,^4,5^ severely limiting their wide applications.^6^ However, the implementation of corrosion protection could be greatly enhanced by a profound understanding of the underlying corrosion mechanisms.^7^ For example, the reaction of metallic Mg with gaseous oxygen (O_2_) and/or water (H_2_O) can form a surface magnesium oxide (MgO) film with a thickness of a few nanometers.^8–11^ Under dry atmospheric conditions, the surface MgO film is protective, while under humid conditions, the surface MgO film can react with H_2_O leading to surface hydroxylation [MgO + H_2_O = Mg­(OH)_2_], resulting in a layered surface film consisting of a Mg­(OH)_2_ cap layer and an MgO sublayer. This layered surface film is not protective at relatively higher humidity, at which water films thicker than three layers possessing similar properties to bulk water will be formed.^12,13^ Such water films tend to cause the dissolution of the quasi-passive MgO/Mg­(OH)_2_ film due to their water solubility [Mg­(OH)_2_(s) = Mg^2+^(aq) + 2OH^–^(aq); MgO(s) + H_2_O­(l) = Mg^2+^(aq) + 2OH^–^(aq)]. The lower solubility of Mg­(OH)_2_ (brucite) compared to MgO (periclase) implies that the surface water film can easily become supersaturated, allowing Mg­(OH)_2_ to precipitate, leading to MgO film thinning and Mg­(OH)_2_ thickening. Since Mg­(OH)_2_ is less protective as it cracks and spalls under the residual stresses arising from oxide growth, it provides little resistance to the transport of H_2_O or O_2_.^12^

Despite considerable studies on magnesium alloy corrosion, the mechanisms are not sufficiently or rationally understood yet.^4–6,14^ To provide models for the prediction and control of corrosion in an engineering context, corrosion experiments are usually designed to elucidate certain aspects of the process. Since corrosion experiments are largely influenced by a corrosion environment including material components, different and contradictory results can be obtained. Although combining the estimated corrosion rate with the detected material microstructure, crystallinity, local composition, surface chemistry, reactivity, corrosion products, or any surface film can give insights into the corrosion reaction,^15^ the mechanisms are more macroscopic than microscopic in nature.

In addition to experiments, theoretical studies have been applied to explore the corrosion mechanism of magnesium in aqueous solution,^7,16–23^ including water splitting [2Mg* + H_2_O → Mg*H + Mg*OH, Volmer pathway], hydrogen evolution [2Mg*H → 2Mg* + H_2_, Tafel pathway; or H_2_O + Mg*H → Mg*OH + H_2_, Heyrovský-like pathway], and OH dissociation [Mg*OH + Mg* → Mg*O + Mg*H]. The Volmer–Heyrovský pathway has been found to be energetically more favorable than the Volmer–Tafel pathway and contributes largely to hydrogen evolution at cathodic and anodic overpotentials. Based on an original mechanistic surface kinetic DFT model, Yuwono et al.^7^ studied the mechanism of anomalous hydrogen evolution (HE) on anodically polarized Mg and found that the HE rate along with the simultaneous dissolution of Mg increased with increasing potentials, and the governing reaction is pH-dependent and has higher kinetics at lower pH values. Since this model only accounts for anomalous HE within a very narrow window of anodic potentials,^7^ it cannot fully explain the numerous empirical observations of anomalous HE over large anodic overpotentials. Using a first-principles approach, Limmer et al.^19^ studied the effect of dilute alloying on the cathodic reaction on the Mg(0001) surface and found that the surface alloying of Ca, Sc, Y, Ti, and Zr makes H_2_O dissociation more exothermic, while that of Ge, In, Sb, and Sn makes the water dissociation reaction endothermic. They further analyzed the mechanisms of preventing HE and found that alloying with early-period elements (Ca, Sc, Ti, Y, and Zr) prevents HE from the surface by binding the adsorbed hydrogen, whereas alloying with late-period elements (Al, As, Cd, Ga, Ge, In, Si, Sn, Sb, and Zn) prevents local hydrogen recombination by repelling adsorbed hydrogen. Nezafati et al.^20^ studied the alloying effects of Al, Zn, Ca, and Y on water molecule adsorption on Mg-based slab systems and found that the alloying of Ca and Y on Mg(0001) strengthens water adsorption, while that of Al and Zn weakens water adsorption. Pang et al.^24^ found that the alloying of Zn and Al strengthens water adsorption on the nearby Mg sites, while H_2_O adsorbs most stably on top of the Ce dopant site. Ng et al.^23^ investigated oxygen evolution (OR) and HE in magnesium corrosion with DFT and found that on pristine and alloyed Mg, both HE and OR contributed to the overall cathodic current density; however, HE contributed much more significantly than OR, the OR contribution was limited by mass transport and would diminish over time. The Pourbaix diagram, a useful tool in the analysis of the fundamental aspects of surface corrosion, was also generated using first principals calculations for understanding the magnesium corrosion process in aqueous solution at the atomic scale (mainly electrochemical corrosion) and explaining the reaction mechanism from an electronic perspective .^17^

Although atmospheric corrosion accounts for more failures in terms of cost and tonnage than any other type of material degradation,^4^ surprisingly very little experimental work has been devoted to the atmospheric corrosion of magnesium as compared to electrochemical corrosion in aqueous solutions. Since most magnesium materials are exposed to atmospheric environments during practical applications rather than being fully immersed in aqueous solutions, understanding their atmospheric corrosion is irreplaceably important, even if the rate is considerably lower than that of electrochemical corrosion in aqueous solutions.^4,25^ Atmospheric corrosion of magnesium materials causes a series of specific failure problems, such as localized corrosion, microgalvanic corrosion, and spallation of corrosion products, which directly limit the reliability and service life of their engineering applications. Studying the atmospheric corrosion of magnesium alloys through exposure experiments including marine, urban, rural, and laboratory atmosphere environments revealed much more complicated mechanisms influenced by many factors, especially atmospheric factors such as temperature, relative humidity, gas composition, and pollutants in the air.^25,26^ Therefore, it is necessary to conduct in-depth research into the atmospheric corrosion mechanisms of Mg, thereby providing a basis for protecting magnesium materials under various application conditions.

In addition to experimental studies, theoretical studies also mainly focused on electrochemical corrosion and paid less attention to atmospheric corrosion. There are many aspects that need to be investigated, such as the thermodynamics and kinetics of water adsorption and reaction on magnesium surfaces under atmospheric conditions, the influence of substances in the air (like O_2_ CO_2_, SO_2_), and alloy techniques on surface reactions. In our previous work,^27^ we systematically computed the surface evolution of the Mg(0001) surface with increasing O_2_ exposure using DFT and AIMD simulations and gave a comprehensive understanding of the initial oxidation film formation and film thickening at the atomic scale and revealed the formation of surface peroxides. In this work, we applied periodic density functional theory to study the surface reaction of H_2_O with Mg(0001) and the surface evolution under atmospheric conditions, along with the influence of oxygen on the reaction of H_2_O with Mg(0001). Nevertheless, it should be noted that magnesium surfaces undergo rapid oxidation in a real atmospheric environment containing O_2_, making the probability of direct contact between clean metal surfaces and H_2_O extremely low. Therefore, investigating clean Mg(0001) surfaces is primarily limited to ultrahigh vacuum model systems within the field of surface science to establish a fundamental framework for the intrinsic interaction between H_2_O and magnesium metal. This helps to clarify the basic energy barriers and paths of water molecule adsorption, dissociation, and hydrogen diffusion on the ideal metal surface by excluding complex interface factors and provides indispensable benchmark data and mechanism comparison baselines for understanding more complex real corrosion processes. Since magnesium surfaces are covered by oxide films in atmospheric environments , and oxide films can rupture and expose fresh metal substrates in scenarios such as mechanical scratches, wear failure, and local plastic deformation, we further analyzed the interaction of oxidized surfaces with water for understanding water adsorption, dissociation, and cathodic reaction kinetics on oxidized magnesium surfaces in real atmospheric environments. By comparing both clean and oxidized surfaces, we can evaluate the passivation effect of oxide layers on water molecule activity and reveal the transition of corrosion control steps from surface reactions to diffusion mass transfer.

## 2 Models and Methods

All calculations were performed based on the periodic slab model using the plane wave-based density functional theory method implemented in the Vienna Ab initio Simulation Package (VASP).^28–30^ The projected augmented wave method (PAW)^31,32^ was used to describe the interaction between electrons and ions. The electron exchange and correlation energies were calculated within the generalized gradient approximation method (GGA) using the Perdew–Burke–Ernzerhof (PBE) functional.^33^ The following PAW pseudopotentials were employed with the valence electron configurations as indicated: Mg (3s^2^), O (2s^2^ 2p^4^), and H (1s^1^). Dispersion correction using the D3 parameter of Grimme^34^ was included for all calculations. All simulations were performed using a 2 × 2 × 1 gamma-centered grid of k-points. The plane-wave expansion was limited by a cutoff energy of 520 eV. Geometry optimization was converged until the forces acting on the atoms were smaller than 0.03 eV/Å, whereas the energy threshold defining self-consistency of the electron density was set to 10^–5^ eV. The climbing image nudged elastic band (CI-NEB) method^35,36^ and improved dimer method (IDM)^37,38^ were used to find the transition state, and frequency analysis was carried out to characterize authentic transition states with only one imaginary frequency.

All structural optimizations and electronic energy calculations were performed at 0 K, and the reported adsorption energies included zero-point energy (ZPE) correction (unless otherwise specified). To evaluate the thermodynamic feasibility of the reaction pathways at finite temperatures, Gibbs free energy changes (Δ*G*) were computed. All frequency calculations were performed on the optimized structures with a displacement of 0.015 Å. For adsorbed species, only adsorbates and the interacting surface atoms were allowed to relax during frequency calculations. The thermal corrections were obtained using the VASPKIT code^39^ (VASPKIT Standard Edition 1.4.0) at *T* = 298 K. For adsorbed molecules, the *pV* contribution to the translational degrees of freedom was neglected (i.e., *H* = *U*). To avoid unphysically large entropy contributions from very low-frequency modes, all frequencies below 50 cm^–1^ were raised to 50 cm^–1^. The zero-point energy (ZPE), thermal correction to internal energy (*U*_corr_), enthalpy (*H*_corr_), and Gibbs free energy (*G*_corr_) were then derived. The Gibbs free energy of each species at temperature *T* is given by *G*(*T*) = *E* + *G*_corr_. For gas-phase molecules (e.g., H_2_, H_2_O), the same procedure was applied using the ideal-gas approximation to include translational, rotational, and vibrational contributions, with the reference state set to 1 atm at 298.15 K.

Projected densities of states (PDOS) were calculated based on the equilibrium structures using the VASP code. To obtain accurate electronic structures, a nonself-consistent projection calculation was performed with a denser k-mesh of 15 × 15 × 1 after structural optimization and a static self-consistent-field (SCF) calculation. The resulting DOSCAR and PROCAR files were postprocessed using VASPKIT^39^ to extract the total and partial DOS. The VASPKIT code was also used to align the Fermi level to 0 eV. To elucidate the chemical bonding characteristics, Crystal Orbital Hamilton Population (COHP) analysis was performed using the LOBSTER package.^40^ A separate self-consistent VASP calculation was conducted as a prerequisite for LOBSTER, ensuring that the WAVECAR file was retained. Subsequently, LOBSTER was executed to project the plane-wave results onto a local orbital basis (Löwdin orthogonalization), generating the COHP curves for selected bonds.

In this study, adsorption energy for *n* × H, *n* × OH, *n* × H_2_O on the Mg(0001) surface is defined in eqs 1–3, where $E_{H_{n}/slab}$ , $E_{{\left(\mathrm{OH}\right)}_{n}/\mathrm{slab}}$ , and $E_{{\left(H_{2}O\right)}_{n}/slab}$ are the total energies of the slab with adsorbed *n* × H atoms, *n* × OH atoms, and *n* × H_2_O molecules, respectively. *E*_slab_ is the total energy of the bare slab, *E*(H_2_) is the total energy of a free H_2_ molecule in the gas phase, and *E*(H_2_O) is the total energy of a free H_2_O molecule in the gas phase.

1 $$E_{ad}\left(H_{n}\right)=E_{H_{n}/slab}-E_{slab}-\frac{n}{2}E_{H_{2}}$$

2 $$E_{ad}\left({\left(\mathrm{OH}\right)}_{n}\right)=E_{{\left(\mathrm{OH}\right)}_{n}/\mathrm{slab}}-E_{slab}-n\left(E_{H_{2}O}-\frac{1}{2}E_{H_{2}}\right)$$

3 $$E_{ad}\left({\left(H_{2}O\right)}_{n}\right)=E_{{\left(H_{2}O\right)}_{n}/slab}-E_{\mathrm{slab}}-nE_{H_{2}O}$$

In addition, average adsorption energy (adsorption energy per adsorbate, eq 4) and stepwise adsorption energy (eqs 5–7) were calculated, where $E_{X_{n}/slab}$ and $E_{X_{n+1}/slab}$ are the total energies of the slab with *n* × *X* and (*n* + 1) × *X* adsorption, respectively. *X* indicates the adsorbate (*X* = H, OH, or H_2_O).

4 $${\mathrm{E̅}}_{ad}\left(X\right)=\frac{E_{ad}\left(X_{n}\right)}{n}$$

5 $$\Delta E_{ad}\left(H\right)=E_{H_{n+1}/slab}-E_{H_{n}/slab}-\frac{1}{2}E_{H_{2}}$$

6 $$\Delta E_{ad}\left(\mathrm{OH}\right)=E_{{\left(\mathrm{OH}\right)}_{n+1}/slab}-E_{{\left(\mathrm{OH}\right)}_{n}/slab}-\left(E_{H_{2}O}-\frac{1}{2}E_{H_{2}}\right)$$

7 $$\Delta E_{ad}\left(H_{2}O\right)=E_{{\left(H_{2}O\right)}_{n+1}/slab}-E_{{\left(H_{2}O\right)}_{n}/slab}-E_{H_{2}O}$$

Due to the special nature of water forming hydrogen bonding and clustering, the energy of hydrogen bonding (eq 8) and the interaction energy of the water-cluster with the metal surface (eq 9) were also computed, where $E_{{\left(H_{2}O\right)}_{n}}$ is the gas-phase total energy of the (H_2_O)_*n*_ cluster from their coadsorbed states.

8 $$E_{H‐bond}=E_{{\left(H_{2}O\right)}_{n}}-nE_{H_{2}O}$$

9 $$E_{w-m}=E_{{\left(H_{2}O\right)}_{n}/\mathrm{slab}}-E_{slab}-E_{{\left(H_{2}O\right)}_{n}}$$

In our calculations, we took the same *p*(4 × 4) Mg (0001) surface model as in our previous work (Figure 1),^27^ i.e., a six-layer slab model with the bottom three layers frozen and the upper three layers along with adsorbates freely relaxed, in which a 15 Å vacuum gap was created between the upper-most and bottom-most layers. As shown in Figure 1, the top three layers allowed to relax are clearly distinguished by color ranging from light to dark. The surface layer has face-centered cubic (*fcc*), hexagonal close-packed (*hcp*), top, and bridge (*bri*) sites for adsorption. The first sublayer has a tetrahedral site under the top site (TUT1), a tetrahedral site under the *hcp* site (TUH1), and an octahedral site (Oct1). The second sublayer has a tetrahedral site under the top site (TUT2), a tetrahedral site under the *hcp* site (TUH2), and an octahedral site (Oct2).

**Figure 1:**
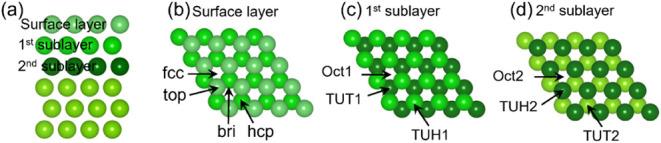
(a) Side view of the surface layer, the first sublayer, and the second layer; (b) top view and adsorption sites of the surface layer; (c) top view and adsorption sites of the first sublayer (by removal of the surface layer); (d) top view and adsorption sites of the second sublayer (by removal of the first two layers) of the *p*(4 × 4) Mg(0001) slab model (reprinted with permission under the terms of the CC BY license from ref 27. Copyright 2025 American Chemical Society).

## 3 Results and Discussion

### 3.1 H_2_O-Dissociative Adsorption on Mg(0001)

As H_2_O molecules finally decompose into H, O, and OH, the adsorption of H_2_O, OH, and H on the Mg(0001) surface is computed first, while that of O is taken from our previous work for comparison.^27^ The adsorption configuration and energy for 1 × H atom, 1 × OH group, and 1 × H_2_O molecule are shown in Figure 2.

**Figure 2:**
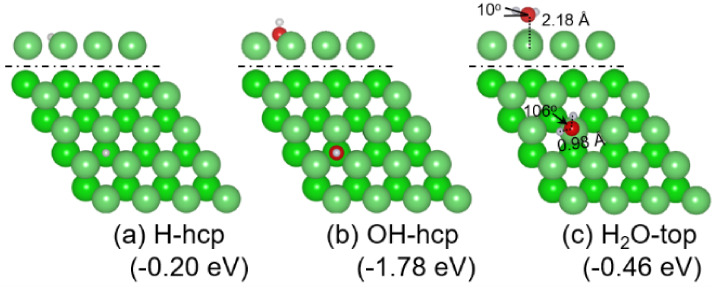
Most stable adsorption configuration (top: side view of the first layer, bottom: top view) and adsorption energy: (a) H, (b) OH, and (c) H_2_O (Mg/green; O/red; H/white).

For the adsorption of an H atom at the lowest coverage (1/16 ML), all of the adsorption sites on the surface and under the first and second subsurface sites are considered, and the corresponding adsorption energies and relative parameters are shown in Table S1. On the surface layer, *hcp* and *fcc* are the stable sites, and the corresponding adsorption energies are −0.20 and −0.16 eV, respectively, indicating a rather weak interaction between the H atom and the Mg(0001) surface. The adsorption energy of an H atom under the subsurface sites is nearly zero or positive, suggesting that H prefers to adsorb on the surface rather than under the subsurface sites. Among these sites, *hcp* is the most stable for one H adsorption (Figure 2a) and the adsorbed H atom is negatively charged (−0.97 e).

Next the number of adsorbed H atoms is increased steadily (Figure S1 and Table S2). Up to 1 ML (16 × H) coverage, the *fcc* sites become more stable than the *hcp* sites, the adsorbed H atoms are aggregated, and the average adsorption energy increases steadily, such as from −0.21 eV for 2 × H adsorption to −0.25 eV for 16 × H adsorption, and such relationship can be clearly seen in the stepwise adsorption energy, such as from −0.22 eV for 2 × H adsorption to −0.36 eV for 16 × H adsorption, indicating enhanced interaction among these aggregately adsorbed H atoms. At 2 ML (32 × H) coverage (adding 16 × H on *hcp* sites based on 16 × H on *fcc* sites), the H atoms on *hcp* sites move to TUH1 sites (Figure S2a), and finally, there are 16 × *fcc* H and 16 × TUH1 H, and the adsorption energy is −8.06 eV (−0.25 eV for each H). At 3 ML (48 × H) coverage (16 × *fcc* H, 16 × TUH1 H, and 16 × oct1 H, Figure S2b), the adsorption energy is −10.10 eV (−0.21 eV for each H). It is noted that the adsorption of H atoms is totally different from that of O atoms; for example, the adsorption of O atoms prefers the subsurfaces at the tetrahedral site under the *hcp* site and the octahedral site.

Different from the adsorption of H, the adsorption of OH on the Mg(0001) surface needs to consider not only possible sites but also the H–O orientation. Based on the calculated adsorption energies, relative parameters, and adsorption configurations (Figure S3 and Table S3), OH also prefers to adsorb on the surface rather than under the subsurface, and the most stable adsorption site is the *hcp* site with O–H perpendicular to the surface in which O interacts with Mg atoms with an adsorption energy of −1.78 eV (Figure 2b) and other adsorption sites either with O–H or H–O perpendicular to the surface are higher in energy. Next the number of adsorbed OH groups is increased steadily. At up to 1 ML (16 × OH) coverage (Figure S4 and Table S4), the OH groups are located at the *hcp* sites with O–H perpendicular to the surface and the adsorbed OH groups form clusters, and the average adsorption energy increases steadily such as from −1.82 eV for 2 × OH to −1.93 eV for 16 × OH, and this relationship can be emphasized by the stepwise adsorption energy, such as from −1.86 eV for 2 × OH to −2.15 eV for 16 × OH, indicating enhanced interaction among these aggregately adsorbed OH groups. It is noted that even at 1 ML coverage, no hydrogen bonding is formed among these adsorbed OH groups in perpendicular configurations.

As shown in Figure 2c, H_2_O prefers to adsorb on the top site with an adsorption energy of −0.46 eV in which the Mg–O distance is 2.18 Å, the tilt angle is about 10°, the H–O–H angle is about 106°, and the O–H bond length is 0.980 Å, indicating a slight change compared to a free H_2_O molecule in the gas phase (i.e., the H–O–H angle is 105° and the O–H bond length is 0.972 Å). Bader charge analysis (Figure S5) reveals a directional charge transfer (0.13 e^–^) upon adsorption from the surface to the adsorbed H_2_O molecule. The PDOS reveals that the main interaction between the adsorbed H_2_O and the Mg surface comes from the frontier orbitals of the H_2_O molecule (Figure 3), i.e., the out-of-planar (1b_1_) and the in-planar (3a_1_) lone pairs,^41^ which are shifted to lower energy based on adsorption. The same is also shown by the COHP analysis.

**Figure 3:**
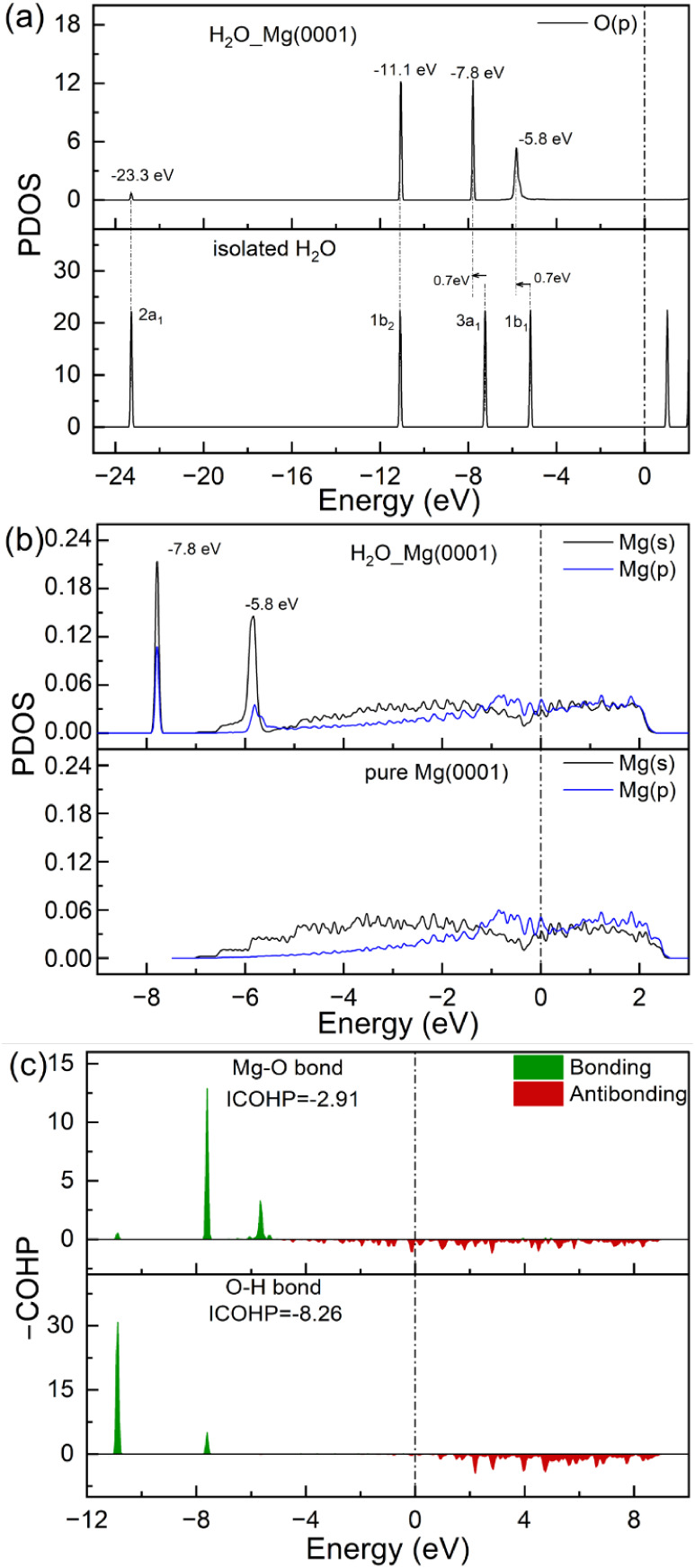
PDOS plots of the O atom of H_2_O adsorbed on the Mg(0001) surface and orbital energy diagram for gas-phase H_2_O (a); PDOS plots of the Mg atom which interacts with the H_2_O molecule and on the pure Mg(0001) surface (b); COHP of the Mg–O bond and H–O bond (ICOHP is also listed) for water adsorption on Mg(0001) (c). The Fermi level is zero, and the isolated H_2_O levels in (a) have been shifted so that the 2a1 peaks in (a) coincide.

With the increase in the adsorbed H_2_O molecules on Mg(0001), aggregated (H_2_O)_*n*_ will form due to intermolecular hydrogen bonding. We used stepwise adsorption energy to find the most stable adsorption configurations of (H_2_O)_*n*_, i.e., one H_2_O molecule was added onto the previous one with the most stable adsorption configuration. The most stable adsorption configurations of (H_2_O)_*n*_ (*n* = 1–8) are given in Figure 4 and the relevant energies and bond parameters are listed in Table 1.

**Figure 4:**
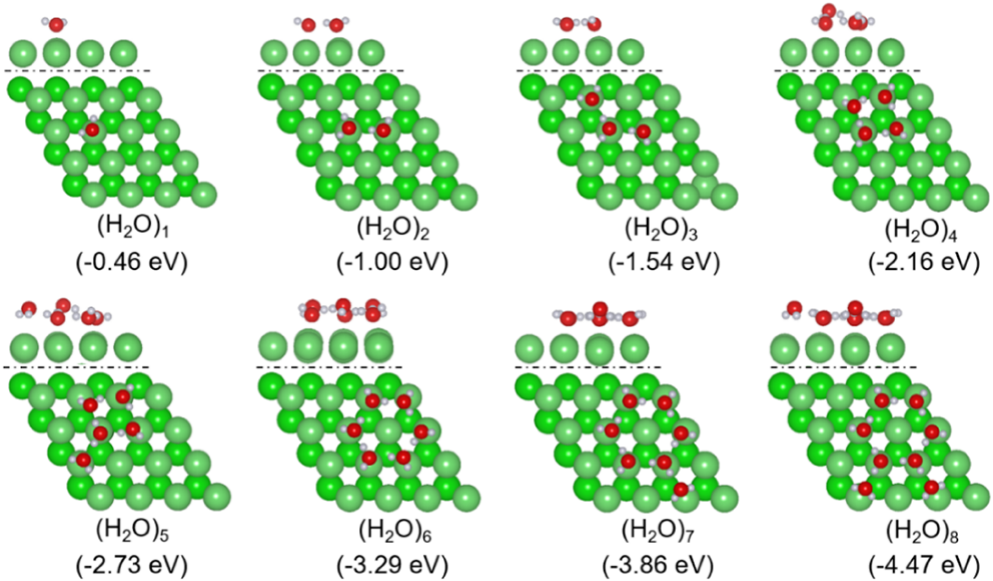
Most stable adsorption configuration (top: side view of the first layer, bottom: top view) and adsorption energy of (H_2_O)_*n*_ clusters on Mg(0001) surface (Mg/green; O/red; H/white).

**Table 1:** Adsorption Energy (*E*_*ad*_, eV/eq 3), Average Adsorption Energy ( ${\mathrm{E̅}}_{ad}$ , eV/eq 4), Stepwise Adsorption Energy (Δ*E*_*ad*_, eV/eq 7), Hydrogen-Bonding Energy (*E*_H‑*bond*_, eV/eq 8), Water–Metal Interaction (*E*_*w*–_ *_m_*, eV/eq 9), Mg–O Distance (*d*_O–Mg_, Å), and H-Bonding Distances (*d*_H‑*bond*_, Å) of (H_2_O)_*n*_ Clusters on Mg(0001) Surface

(H_2_O)_*n*_	*E*_*ad*_	${\mathrm{E̅}}_{ad}$	Δ*E*_*ad*_	*E*_*w*–_ *_m_*	*E*_H‑*bond*_	*d*_O–Mg_	*d*_H‑*bond*_
1	–0.46	–0.46		–0.47		2.178	-
2	–1.00	–0.50	–0.54	–0.98	–0.06	2.337, 2.158	1.881
3	–1.54	–0.51	–0.54	–1.36	–0.23	2.364, 2.310, 2.166	1.866, 1.864
4	–2.16	–0.54	–0.62	–1.42	–0.80	3.597, 2.243, 2.278, 2.277	1.986, 1.875, 1.732, 1.582
5	–2.73	–0.55	–0.57	–1.93	–0.90	3.615, 2.262, 2.261, 3.271, 2.183	1.983, 1.856, 1.679, 1.688, 1.632
6	–3.29	–0.55	–0.55	–2.08	–1.49	3.309, 2.202, 3.367, 2.204, 3.427, 2.212	1.899, 1.612, 1.904, 1.607, 1.873, 1.609
7	–3.86	–0.55	–0.58	–2.55	–1.41	2.352, 2.346, 2.317, 2.261, 2.322, 2.337, 3.356	1.821, 1.830, 1.816, 1.736, 1.944, 1.859, 1.692
8	–4.47	–0.56	–0.61	–3.14	–1.46	2.341, 2.336, 2.323, 2.242, 2.244, 2.312, 3.390, 3.366	1.849, 1.830, 1.808, 1.730, 1.815, 1.854, 1.684, 1.693

For 2 × H_2_O coadsorption, both H_2_O molecules prefer to adsorb on the top sites with one longer (2.337 Å) and one shorter (2.158 Å) Mg–O distance compared to that of single H_2_O adsorption (2.178 Å) as well as hydrogen-bonding (1.881 Å) between the two adsorbed H_2_O molecules, and the adsorption energy is −1.00 eV, which is more than twice the adsorption energy of a single H_2_O molecule (−0.46 eV). To differentiate this enhanced interaction, the gas-phase single-point energies of the 2 × H_2_O from their coadsorbed states were computed. It is found that the adsorption energy of this dimeric structure is −0.98 eV, indicating that the adsorption energy comes mainly from the O–Mg interaction, and the contribution from hydrogen-bonding is rather small.^21^

For 3 × H_2_O coadsorption in a bent shape, the middle H_2_O adsorbs on the top site, providing one H atom and one O atom for hydrogen bonding and the O–Mg distance is 2.310 Å, and the O–Mg distances of the other two H_2_O molecules are 2.364 and 2.166 Å, respectively. The lengths of the two formed hydrogen bonds are 1.866 and 1.864 Å, shorter than that of the coadsorption of 2 × H_2_O (1.881 Å). The total adsorption energy is −1.54 eV, three times larger than that of a single H_2_O adsorption. The average adsorption energy is −0.51 eV and the stepwise adsorption energy is −0.54 eV, close to that for the second H_2_O stepwise adsorption. As the number of adsorbed H_2_O increases, H_2_O molecules aggregate together through hydrogen bonding to form clusters, and the adsorption becomes more stable as the hydrogen bonding increases, as indicated by the increased average and stepwise adsorption energies. It is also noted that such an increase in stability comes from the hydrogen bonding within the cluster, and this is because some H_2_O molecules have very long O–Mg distances. For the hexagonal cluster (H_2_O)_6_, for example, the O–Mg distances alternate, i.e., three shorter (2.202, 2.204, and 2.212 Å) and three longer (3.309, 3.367, and 3.427 Å). At the same time, there are six alternating hydrogen bonds, three longer (1.899, 1.904, and 1.873 Å) and three much shorter (1.612, 1.607, and 1.609 Å). Therefore, not only the O–Mg interaction but also hydrogen bonding determines the structures and stability of the adsorbed water clusters, in agreement with the reported results.^22,42^

### 3.2 H_2_O Dissociation on Mg(0001)

#### 3.2.1 Monomolecular H_2_O Dissociation

The potential Gibbs free energy surface of the dissociative adsorption of one water molecule on the Mg(0001) surface under atmospheric conditions [298 K, *p*(H_2_O) = 1 atm, *p*(H_2_) = 1 atm] is shown in Figure 5. The structural parameters of the initial, transition, and final states are shown in Table S5, and the reaction barriers, reaction energies, and relevant structural parameters of the transition state are listed in Table S6. Starting with H_2_O adsorbed on the top site, the first O–H dissociation has a Gibbs free energy barrier of 0.45 eV and is exergonic by 1.54 eV. In the transition state (**TS1**), the breaking distance of the O–H bond is 1.300 Å. In the final state, both OH and H are located at the *hcp* sites.

**Figure 5:**
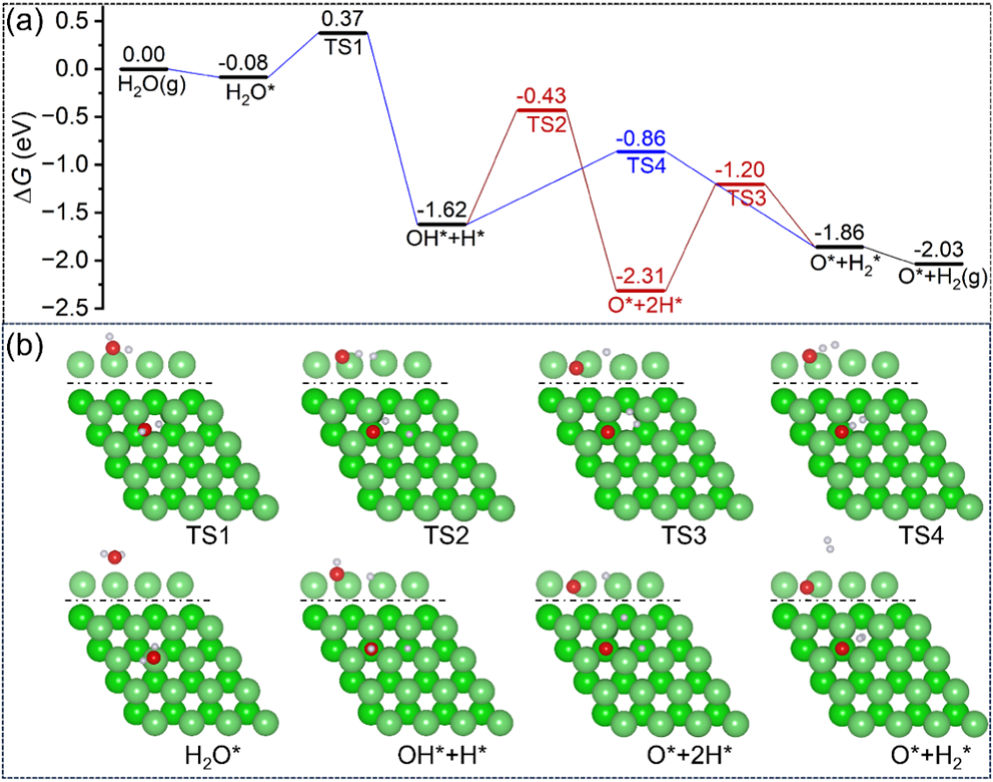
(a) Potential Gibbs free energy surface and (b) corresponding adsorption configuration (top: side view of the first layer, bottom: top view; Mg/green; O/red; H/white) for single H_2_O-dissociative adsorption on the Mg(0001) surface (* for surface species, g for gas-phase species).

From the coadsorbed OH and H, there are two possible dissociation pathways, one is the direct OH* dissociation and the other is the surface H* -associated OH* dissociation. In the direct OH* dissociation, the dissociation has a Gibbs free energy barrier of 1.19 eV and is exergonic by 0.69 eV, and the breaking O–H distance (**TS2**) is 1.413 Å. The subsequent formation of adsorbed H_2_* from two surface H* has a Gibbs free energy barrier of 1.11 eV and is endergonic by 0.45 eV, and in the transition state (**TS3**), the forming H–H distance is 1.210 Å. This step is also called Tafel hydrogen evolution.

In the H*-associated OH* dissociation, the surface H* can interact with the surface OH* to form surface H_2_* and O*, and in the transition state (**TS4**), the breaking of the O–H distance is 1.239 Å and the forming of the H–H distance is 1.035 Å. This step has a lower Gibbs free energy barrier of 0.76 eV and is exergonic by 0.24 eV, and this is also the so-called Heyrovský hydrogen evolution, which is more favorable kinetically than the Tafel hydrogen evolution by 0.35 eV, and this kinetic advantage comes from the fact that surface H* is negatively charged, while the H of surface OH* is positively charged (Figure S6), and this electrostatic attractive interaction lowers the barrier. The desorption of H_2_* is exergonic by 0.17 eV.

The energetic and structural differences between **TS1** and **TS2** in the first and second O–H dissociation as well as between **TS3** and **TS4** in H–H bond formation can also be explained by the ICOHP analysis (Figures S7–S8). For example, **TS1** with a shorter O–H bond has stronger bonding and is lower in energy than **TS2** with a longer O–H bond. The same is also found for the H–H bond formation; i.e., **TS4** with a shorter H–H bond has stronger bonding and is lower in energy than **TS3** with longer H–H bonding.

Based on the potential Gibbs free energy surface, it is clear that H_2_O prefers to desorb from the kinetic point of view since the desorption energy is lower than the first O–H dissociation barrier (0.08 vs 0.45 eV); however, H_2_O thermodynamically prefers the dissociative adsorption with the formation of gaseous H_2_ and adsorbed O*.

#### 3.2.2 Dimeric (H_2_O)_2_ Dissociation

Under the same conditions, we computed the dissociation of the adsorbed (H_2_O)_2_ cluster. The relevant structural parameters are listed in Table S7. The reaction barriers and reaction energies as well as relevant structural parameters of the transition state are listed in Table S8. The potential Gibbs free energy surface and the corresponding adsorption configuration are shown in Figure 6.

**Figure 6:**
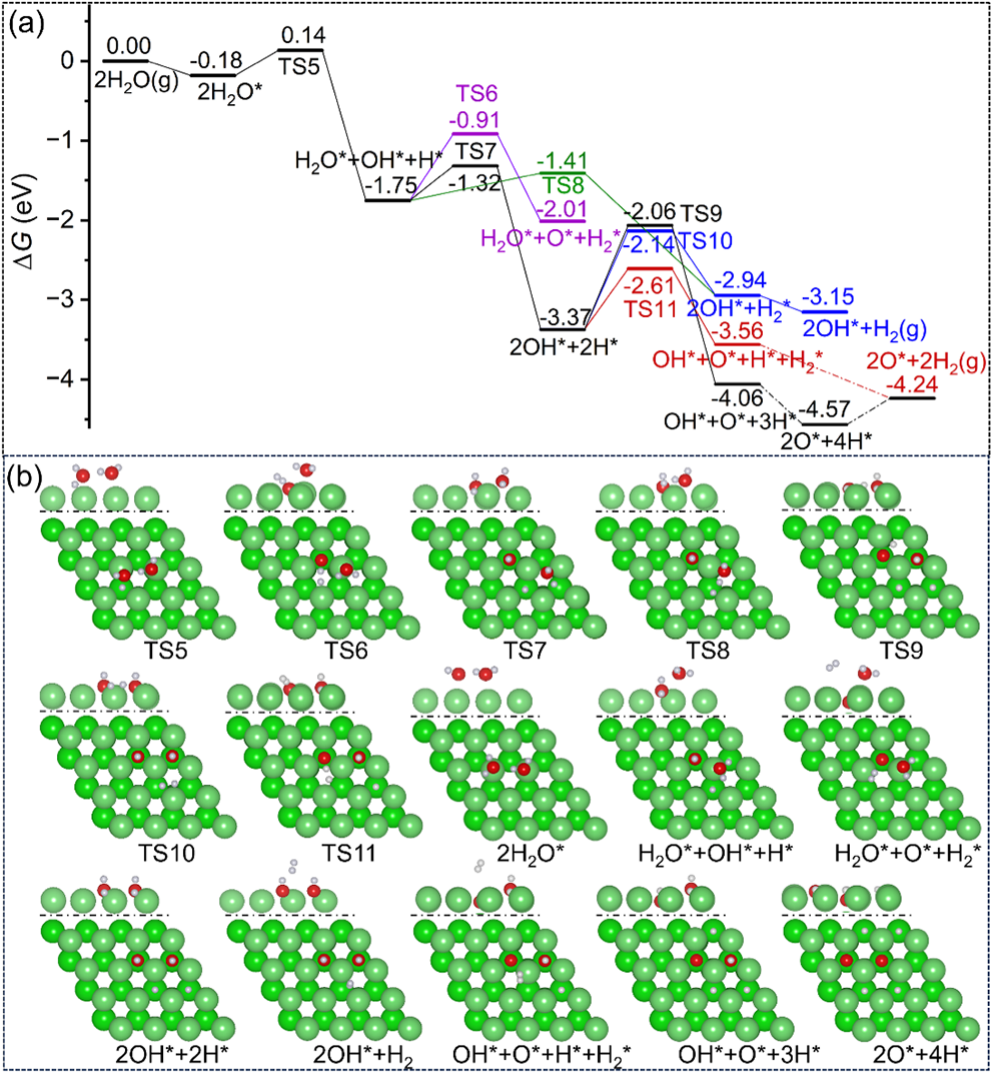
(a) Potential Gibbs free energy and (b) corresponding adsorption configuration (top: side view of the first layer; bottom: top view; Mg/green; O/red; H/white) for water dimer-dissociative adsorption on the Mg(0001) surface (* for surface species, g for gas-phase species).

As found in the one H_2_O dissociation, the first dissociation step (**TS5**) has a low Gibbs free energy barrier of 0.32 eV and is exergonic by 1.57 eV. Next, we computed three subsequent dissociation pathways based on the coadsorbed H_2_O* + OH* + H*, i.e., (*i*) H*-associated OH* dissociation (**TS6**) forming surface H_2_O* and O*, and adsorbed H_2_*, which has a Gibbs free energy barrier of 0.84 eV and is exergonic by 0.26 eV; (*ii*) the direct dissociation of the second H_2_O* (**TS7**) forming surface 2OH* + 2H*, which has a Gibbs free energy barrier of 0.43 eV and is exergonic by 1.62 eV; and (*iii*) H*-associated H_2_O* dissociation (**TS8**) forming surface 2OH* and adsorbed H_2_*, which has a Gibbs free energy barrier of 0.34 eV and is exergonic by 1.19 eV. Although **TS8** is slightly more kinetically favored than **TS7** (0.09 eV), the back reaction of 2OH* + H_2_* forms 2OH* + 2H* with a rather lower barrier of 0.80 eV (**TS10**) and is exergonic by 0.43 eV. Based on the coadsorbed 2OH* + 2H*, the H*-associated OH* dissociation (**TS11**) will lead to the formation of surface O* and adsorbed H_2_*, which has a Gibbs free energy barrier of 0.76 eV, and further OH dissociation will lead to surface oxidation, the same as found for one H_2_O-dissociative adsorption. On the contrary, the direct OH dissociation (**TS9**), which has a rather higher Gibbs free energy barrier of 1.31 eV, is not competitve compared to the H*-associated OH* dissociation (**TS11**).

### 3.3 Surface O*-Associated H_2_O Dissociation

Since O_2_ with its very strong dissociative adsorption energy (−9.07 eV) can oxidize the Mg(0001) surface at ultralow O_2_ content^27^ as well as H_2_O-dissociative adsorption has a Gibbs free energy barrier and much lower adsorption energy, and water molecules interact more likely with surfaces precovered by oxygen atoms under atmospheric conditions, we further analyzed the interaction of the oxidized surfaces with water for understanding the real atmospheric corrosion. As shown in Figures S9–S11, the adsorption energy of H_2_O on O*, 2O*, and 3O* precovered Mg(0001) surfaces is −0.59, −0.69, and −0.70 eV, respectively, which is stronger than that on the clean surface (−0.46 eV), indicating that the O* atoms on Mg(0001) can enhance water adsorption.

To elucidate the role of preadsorbed oxygen in promoting H_2_O adsorption and dissociation, we analyzed the electronic structure of the surface species. Figure 7a displays the PDOS for the oxygen atoms of H_2_O (denoted as O_w_) and the preadsorbed oxygen (denoted as O_a_), as well as for the Mg atom directly interacting with the water molecule. The COHP analysis for the O_w_–H, Mg–O_w_, and Mg–O_a_ bonds is presented in Figure 7b.

**Figure 7:**
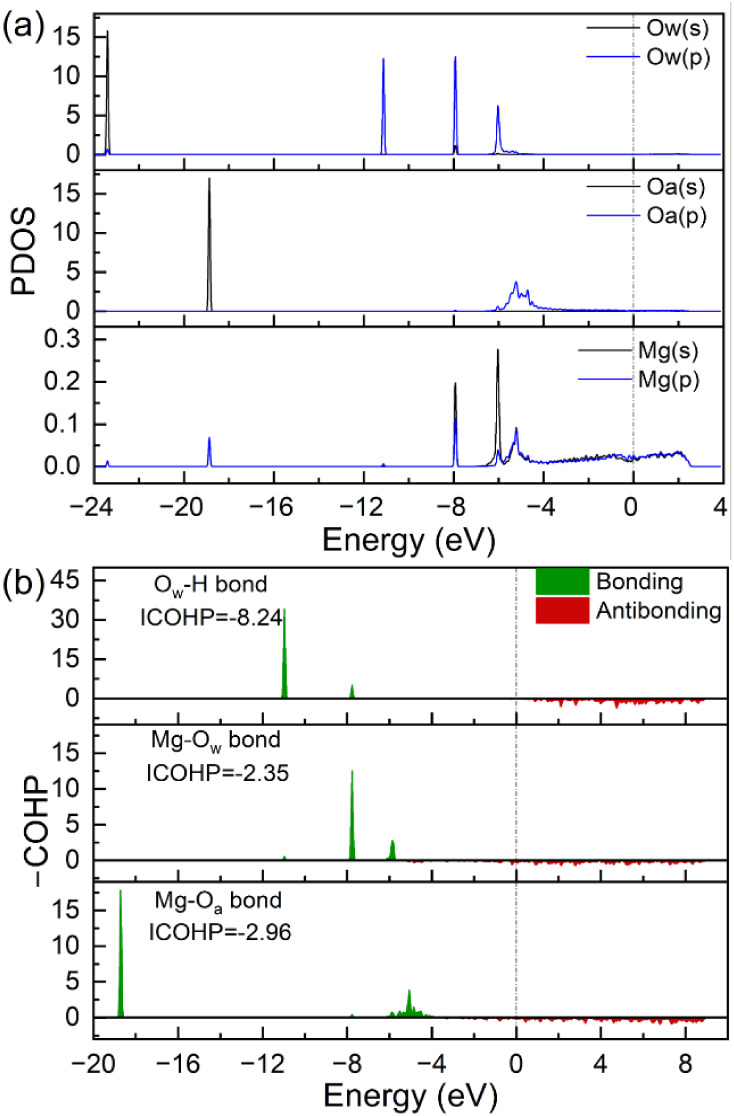
PDOS plots of the O atom of H_2_O (O_w_), precovered O atom (O_a_), and the Mg atom which interacts with the H_2_O molecule (a), and COHP of Mg–O_w_ bond and H–O_w_ bond (b) for water adsorption on Mg(0001).

As shown in Figure 7a, the PDOS of O_w_ closely resembles that of H_2_O adsorbed on clean Mg(0001) (Figure 3 and Figure S12a). However, the PDOS of the O-precovered surface exhibits two new features at – 18.8 and −5.2 eV compared to the clean Mg(0001) case (Figure S12b), originating from the preadsorbed O_a_ atom. This assignment is confirmed by the COHP analysis (Figure 7b): the Mg–O_a_ bond shows pronounced bonding peaks at nearly identical energies (−18.7 eV and −5.1 eV), demonstrating that these hybridized states contribute directly and beneficially to Mg–O_a_ bonding.

As shown in Figure 7a, water adsorption does not change the PDOS of the surface of the O_a_ (Figure S12c). The COHP profiles of the O_w_–H and Mg–O_w_ bonds remain qualitatively similar to those on clean Mg(0001) (Figure S12d and e). Notably, the integrated COHP (ICOHP) values become more positive for both bonds, particularly for Mg–O_w_, indicating that the presence of preadsorbed oxygen slightly weakens the Mg–O_w_ interaction. This suggests that the increased H_2_O adsorption energy may arise from the enhanced electrostatic interaction between adsorbed H_2_O and the positively charged Mg atom. On the other hand, the Mg–O_a_ bond undergoes strengthening upon H_2_O adsorption, as indicated by the more negative ICOHP value for Mg–O_a_ upon H_2_O adsorption. This result is rationalized by Bader charge analysis (Figure S13). Upon H_2_O adsorption, the Mg atom bound to water loses 1.2 e^–^, a substantial increase of 1.0 e^–^ compared to the O precovered surface without H_2_O. However, only a small fraction (0.19 e^–^) of this charge is transferred to the water molecule (specifically to O_w_); this enhances stronger electrostatic interaction between the adsorbed H_2_O and the more positively charged Mg atom.

Starting from the coadsorbed configurations of H_2_O* + O*, H_2_O* + 2O*, and H_2_O* + 3O*, the O*-associated first-step H_2_O dissociation becomes more favorable kinetically than the first H_2_O dissociation directly with the increase of surface O coverage, such as by 0.03, 0.19, and 0.21 eV for 1O*, 2O*, and 3O* (Figures S9–S11), respectively, indicating that the first step should be the dissociative H_2_O adsorption with the formation of the coadsorbed OH* + O*. Based on the coadsorbed OH* + O*, the next step should be the direct dissociation of OH*, followed by either direct or stepwise H_2_ formation and evolution, the same steps as found for H_2_O and (H_2_O)_2_-dissociative adsorption. The results show that O species on Mg(0001) significantly promote water decomposition by lowering the activation barrier for H–OH bond cleavage, similar to their role on the Co(0001) surface.^43^

Based on these results, we computed the dissociative H_2_O adsorption on the fully oxidized surface with two oxygen layers (32O*) preadsorbed on the Mg(0001) surface from our previous result;^27^ with the formation of a surface hydroxyl layer, the change in Gibbs free energy for water-dissociative adsorption is shown in Figure 8 and the adsorption configuration is shown Figure S14. The adsorption energy (*E*_*ad*_) of one H_2_O molecule on the two oxygen layers (32O*) preadsorbed Mg(0001) surface is −1.03 eV, indicating that H_2_O adsorption on this surface is more stable than that on the pure Mg(0001) surface and *n* × O* atoms precovered surface (*n* = 1–3). As shown in Figure 8, there are two pathways for H_2_O molecule dissociation on the 32O* surface. For one H_2_O molecule, the first pathway (black line) is the dissociation assisted by the preadsorbed O* atoms, forming two OH* [31O* + 2OH*) and the second pathway (blue line) is the direct dissociation forming an *fcc* OH* and an *fcc* H* [32O* + OH* + H*], and the latter is more favored thermodynamically than the former by 1.97 eV; this is because the OH* formed at the *hcp* site elevates an O* originally at the TUH1 site to the surface, which makes the structure less stable. The same thermodynamic trend is also found for up to 16 water molecules.

**Figure 8:**
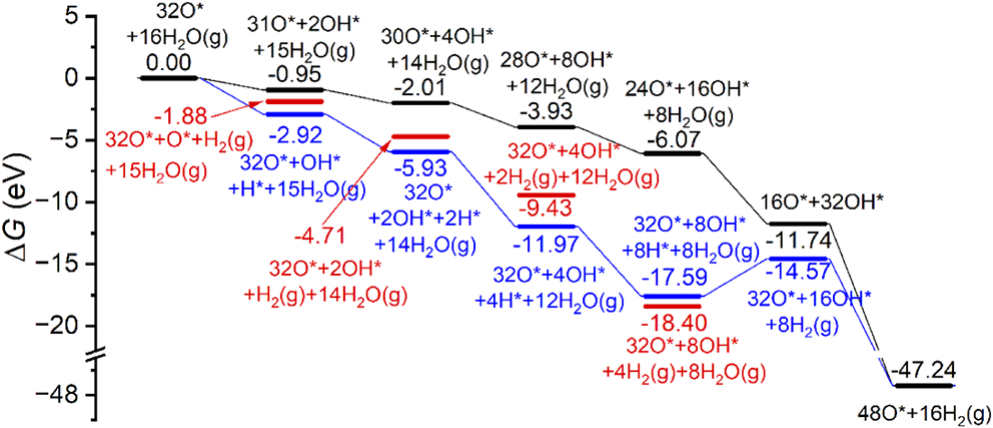
Gibbs free energy for water-dissociative adsorption on 32O preadsorbed Mg(0001) surface (* for surface species, g for gas-phase species).

For the direct pathway for the first H_2_O molecule, the formation of [32O* + OH* + H*] is more favored thermodynamically than the subsequent dissociation with the formation of gaseous H_2_ [32O* + O* + H_2_(g)] by 1.04 eV; this is because tetrahedral sites within the lattice are fully occupied, and the newly formed O* is at the surface *fcc* site. The same is also found for two and four H_2_O molecules; however, for eight H_2_O molecules, surface hydrogen atoms tend to recombine into H_2_ to escape out of the surface into the gas phase. Compared to eight H_2_O molecules dissociating into [32O* + 8OH* + 4H_2_(g)], the direct dissociation of all 16 H_2_O molecules into [32O* + 16OH* + 8H_2_(g)] becomes endergonic by 3.83 eV. For comparison, we computed the structure of O*-assisted dissociation for all 16 H_2_O molecules into [16O* + 32OH*], which is higher in energy than [32O* + 16OH* + 8H_2_(g)] by 2.83 eV. However, the thermodynamically most stable adsorption has the structure of 48O* + 16H_2_(g). All these results reveal the formation of a surface hydroxyl layer at some coverage as thermodynamically stable. Combining this with our previous study on the formation and development of the oxide film on the magnesium surface,^27^ we can see that deeper oxidation can be hindered by the potential barriers of OH* dissociation and O* diffusion into the bulk. The results are consistent with experimental findings,^44^ where surface hydroxyl groups are observed on the thin oxide film formed on the Mg(0001) surface following water dissociation.

## 4 Conclusion

Although metallic magnesium (Mg), the lightest structural metal and one of the most abundant metallic elements, has great technical application potential, its high susceptibility to corrosion from oxidation under atmospheric environments limits its practical uses. To provide insight into the atmospheric corrosion of metallic magnesium surfaces for corrosion protection, we systematically performed DFT computations on the dissociative adsorption mechanism of H_2_O on the Mg(0001) surface.

It is found that H_2_O prefers dissociative adsorption on the Mg(0001) surface kinetically and thermodynamically, forming oxidized islands, which further boost water-dissociative adsorption, and consequently, the reaction rate increases with increasing water exposure, leading to the formation of expanded oxidized islands and a fully covered surface. In addition, the spontaneous dissociation of gaseous O_2_ molecules can also form oxidized islands and promote further water dissociation.

Although the formation of fully oxidized layers is more thermodynamically favorable, the higher activation energy of surface OH* dissociation and the stiffness of the oxidized layers suppress deeper oxidation, forming surface oxidized layers with hydroxyl layers, which will hinder further reaction of O_2_ and H_2_O on the surface under certain conditions. This is consistent with the fact that under atmospheric conditions, the magnesium surface is covered with an oxidized layer and a capping hydroxyl layer. However, this surface structure is attractive to H_2_O molecules, so it can be speculated that under high humidity, water films can be formed on the surface, which will dissolve the surface film. More in-depth research will be needed in the future using kinetic methods to directly simulate the complete dissolution and membrane growth process.

These findings deepen our understanding of atomic-scale corrosion mechanisms in magnesium and provide theoretical guidance for the design of novel corrosion prevention strategies for magnesium alloys. Future work may further investigate the influence of complex atmospheric components such as CO_2_ and Cl^–^ to elucidate the competitive adsorption mechanism of Cl^–^ on the magnesium surface and its critical role in disrupting oxidation layer formation, and investigate the dual role of CO_2_ in modulating the compactness and phase composition of the protective layer, distinguishing between carbonate-induced densification and dissolution.

## Supplementary Material



## Notes

Computational methods, adsorption configuration; adsorption sites, adsorption energy, mean adsorption energy, and stepwise adsorption energy under different coverages; adsorption Gibbs free energy, dissociation barriers and energies, dissociation Gibbs free energy barriers and Gibbs free energies, and bond lengths; potential energy surfaces, Bader charge analysis, COHP curves, and PDOS plots (PDF)
