# Supplementary material for: H2O‑Dissociative Adsorption on Mg(0001) Surface—Corrosion Process under Atmospheric Conditions
Source: ACS Omega. 2026 May 1;11(18):26861–9. doi: 10.1021/acsomega.6c00009 (PMC13177234; doi:10.1021/acsomega.6c00009)
Supplement: Supplementary file 1 [file ao6c00009_si_001.pdf]

H_2O Dissociative Adsorption on Mg(0001) surface –Corrosion Process Under Atmospheric Condition

Yunyan Han,[a, b] and Haijun Jiao[b*]

a. Key Laboratory of Eco-functional Polymer Materials of the Ministry of Education, College of Chemistry and Chemical Engineering, Northwest Normal University, Lanzhou, 730070, China.

b. b. Leibniz-Institut für Katalyse e.V. (LIKAT), Albert-Einstein-Str. 29A, 18059, Rostock, Germany.

[*]Corresponding author.

E-mail: Haijun.Jiao@catalysis.de

Computational Methods

All structural optimizations and electronic energy calculations were performed at 0 K. The adsorption energies involved in the report (unless otherwise specified) are all calculated based on these 0 K electronic energies (E) with Zero-point energy (ZPE) correction. All frequency calculations were performed on the optimized structures with a displacement of 0.015 Å; for adsorbed species, only the adsorbate and the interacted surface atoms were allowed to relax during frequency calculations. To evaluate the thermodynamic feasibility of the reaction pathways at finite temperature, Gibbs free energy changes (ΔG, T = 298 K) were computed. The thermal corrections were obtained using the VASPKIT code (*Computer Physics Communications* **2021,** *267*, 108033) (VASPKIT Standard Edition 1.4.0) at T=298 K. For adsorbed molecules, the *pV* contribution to the translational degrees of freedom was neglected (i.e., H = U). To avoid unphysically large entropy contributions from very low-frequency modes, all frequencies below 50 cm^{-1} were raised to 50 cm^{-1}. The zero-point energy (ZPE), thermal correction to internal energy (U_{corr}), enthalpy (H_{corr}), and Gibbs free energy (G_{corr}) were then derived. The Gibbs free energy of each species at temperature T is given by $G(T)$=E+G_{corr}. For gas-phase molecules (e.g., H_2, H_2O), the same procedure was applied, using the ideal-gas approximation to include translational and rotational contributions, correspond to these Gibbs free energies at 298.15 K and the pressure is 1 atm.

Table S1 Adsorption energy (E_{ad}, eV), shortest Mg-H distance ($d_{H\text{-}Mg}$, Å) and Bader charge (δ_H, e^-) of surface, subsurface H atom in Mg(0001) (0.0625 ML).

			surface		1st subsruface			2nd subsurface		
Site	top†	bri*	fcc	hcp	oct1	TUH1*	TUT1	oct2	TUH2	TUT2
E_{ad}	-	-	-0.16	-0.20	-0.00	-	-0.09	0.05	-0.01	0.07
d_{H-Mg}	-	-	1.991	1.983	2.127	-	1.979	2.213	1.974	1.977
δ_H	-	-	-0.97	-0.98	-1.31	-	-1.13	-1.34	-1.13	-1.14

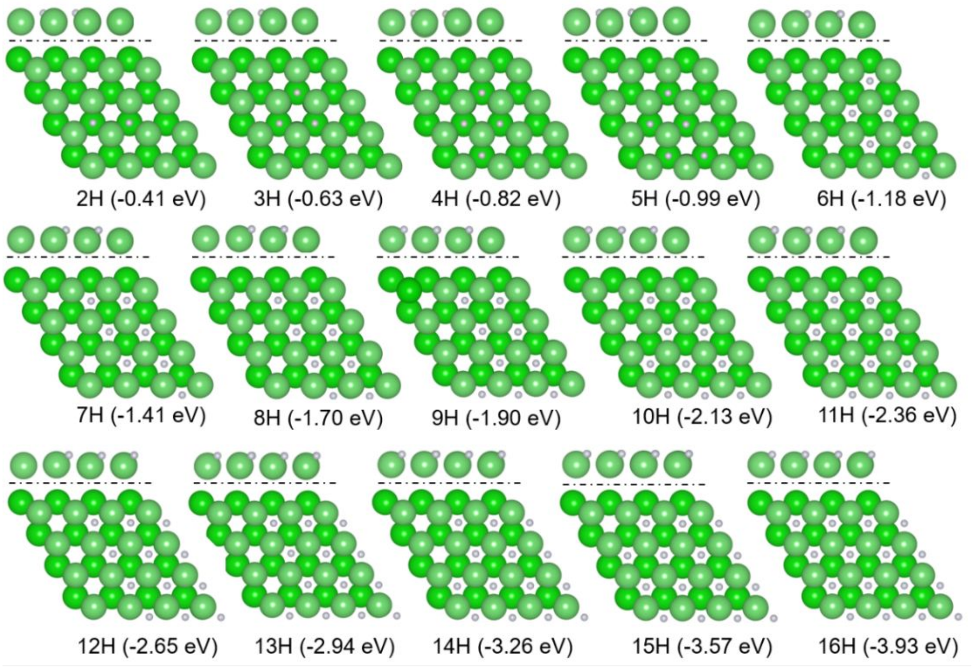

Fig. S1 Most stable adsorption configurations of H atoms (2-16 atoms) on Mg(0001) surface (Mg/green; H/white)

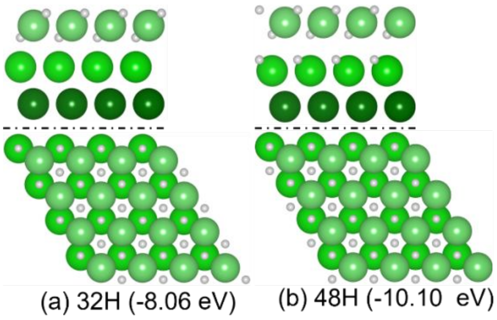

Fig. S2 Adsorption configurations of 32 ◇ H atoms and 48 ◇ H atoms on Mg(0001) surface (Mg/green; H/white)

Table S2 Adsorption sites, adsorption energy (E_{ad}), mean adsorption energy ($\overline{E_{ad}}$), stepwise adsorption energy(δE_{ad}) for the most stable H adsorption configuration on Mg(0001) under different coverages.

Number of H	Coverage (ML)	Site	E_{ad}/eV	$\overline{E_{ad}}$/eV	δE_{ad}/eV
1	0.0625	hcp	-0.20	-0.20	
2	0.125	2×hcp	-0.41	-0.21	-0.22
.3	0.1875	3×hcp	-0.63	-0.21	-0.22
4	0.25	4×hcp	-0.82	-0.20	-0.19
5	0.3125	5×hcp	-0.99	-0.20	-0.17
6	0.375	6×fcc	-1.18	-0.20	-0.19
7	0.4375	7×fcc	-1.41	-0.20	-0.24
8	0.5	8×fcc	-1.70	-0.21	-0.29
9	0.5625	9×fcc	-1.90	-0.21	-0.20
10	0.625	10×fcc	-2.13	-0.21	-0.23
11	0.6875	11×fcc	-2.36	-0.21	-0.23
12	0.75	12×fcc	-2.65	-0.22	-0.28
13	0.8125	13×fcc	-2.94	-0.23	-0.30
14	0.875	14×fcc	-3.26	-0.23	-0.31
15	0.9375	15×fcc	-3.57	-0.24	-0.31
16	1	16×fcc	-3.93	-0.25	-0.36
32	2	16fcc+16TUH1	-8.06	-0.25	-
48	3	16fcc+16TUH1+16oct1	-10.10	-0.21	

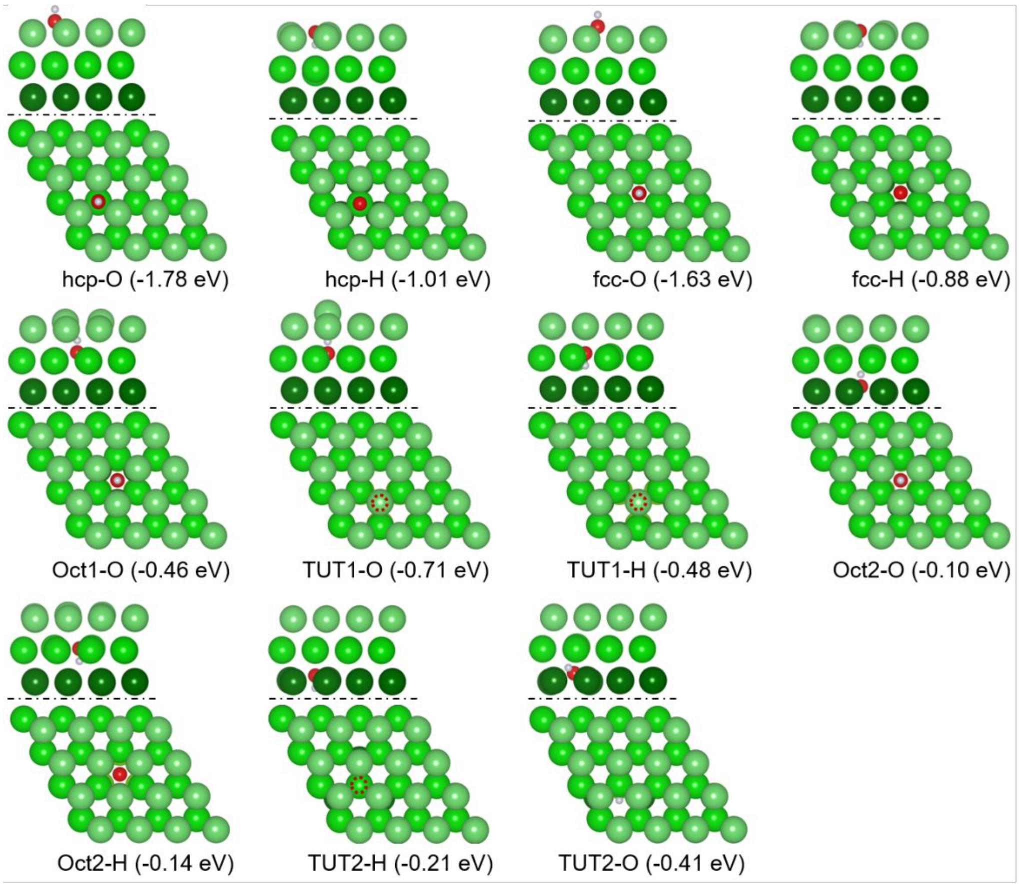

Fig. S3 Adsorption configuration of OH on Mg(0001) surface

Table S3 Adsorption energy and structural data for surface and subsurface OH on Mg(0001) (θ=0.0625).

	surface				1st subsurface			2nd subsurface			
**Site**	**hcp-O**	**hcp-H**	**fcc-O**	**fcc-H**	**oct1-O**	**TUT1-O**	**TUT1-H**	**oct2-O**	**oct2-H**	**TUT2-H**	**TUT2-O**
E_{ad}(eV/atom)	-1.78	-1.01	-1.63	-0.88	-0.46	-0.71	-0.48	-0.10	-0.14	-0.21	-0.41
d_{O-Mg}(Å)	2.096	2.075	2.093	2.059	2.038	2.027	2.102	2.021	2.036	2.091	2.061
d_{H-Mg}(Å)	2.677	2.198	2.703	2.199	2.451	2.321	2.209	2.379	2.285	2.033	2.175
d_{O-H}(Å)	0.978	1.010	0.976	1.019	1.002	0.990	1.018	1.010	1.018	1.017	1.034

d_{H-Mg} is the distance between the H atom and an adjacent Mg atom; d_{O-Mg} is the distance between the O atom and an adjacent Mg atom; d_{O-H} is the distance between the H atom and O of OH species. Other sites which is not listed in the table is either not stable or not local minimum (OH on top site is not stable. OH on bri sites moves to hcp site after optimization, so they are not included in the table)

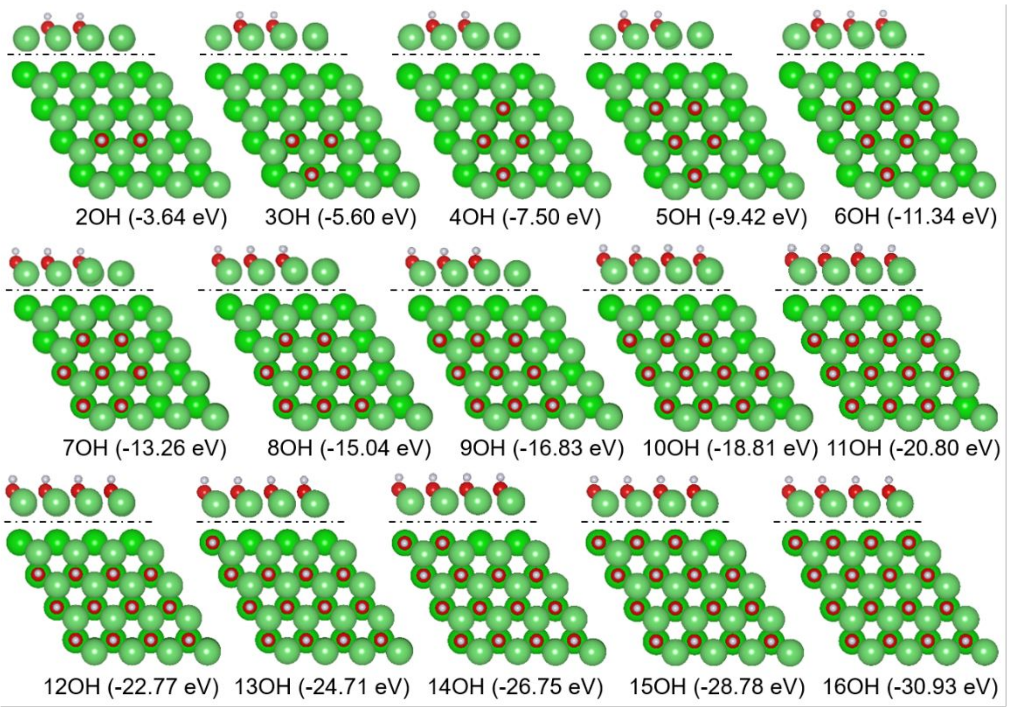

Fig. S4 Most stable adsorption configurations of OH atoms (2-16 atoms) on Mg(0001) surface (Mg/green; O/red; H/white)

Table S4 Adsorption sites, adsorption energy (E_{ad}), mean adsorption energy ($\overline{E_{ad}}$), stepwise adsorption energy(δE_{ad}) for the most stable OH adsorption configuration on Mg(0001) under different coverages. (with ZPE correction)

Number of OH	Coverage (ML)	Site	E_{ad}/eV	$\overline{E_{ad}}$/eV	δE_{ad}/eV
1	0.0625	hcp	-1.78	-1.78	
2	0.125	2×hcp	-3.64	-1.82	-1.87
3	0.1875	3×hcp	-5.60	-1.87	-1.95
4	0.25	4×hcp	-7.50	-1.87	-1.90
5	0.3125	5×hcp	-9.42	-1.88	-1.92
6	0.375	6×hcp	-11.34	-1.89	-1.93
7	0.4375	7×hcp	-13.26	-1.89	-1.92
8	0.5	8×hcp	-15.04	-1.88	-1.77
9	0.5625	9×hcp	-16.83	-1.87	-1.79
10	0.625	10×hcp	-18.81	-1.88	-1.98
11	0.6875	11×hcp	-20.80	-1.89	-2.00
12	0.75	12×hcp	-22.77	-1.90	-1.96
13	0.8125	13×hcp	-24.71	-1.90	-1.94
14	0.875	14×hcp	-26.75	-1.91	-2.04
15	0.9375	15×hcp	-28.78	-1.92	-2.03
16	1	16×hcp	-30.93	-1.93	-2.15

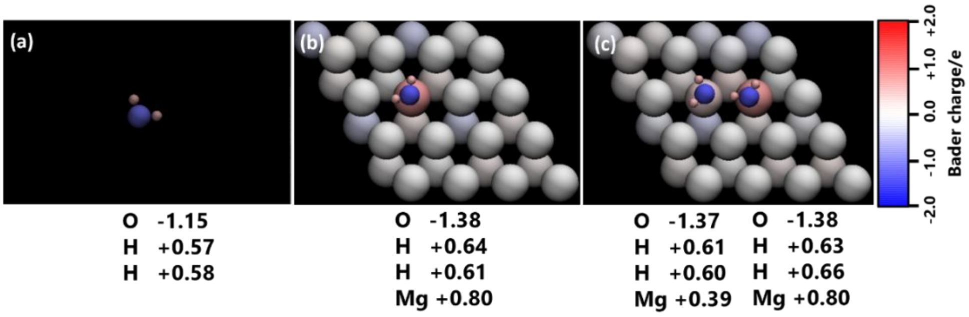

Fig. S5 Bader charge analysis of (a) gas phase H_2O; (b) H_2O adsorbed Mg(0001); (c) $2H_2O$ adsorbed Mg(0001)

Table S5 Adsorption energy (E_{ad}, eV), adsorption Gibbs free energy (G_{ad}, eV) and shortest distances (d, Å) of the IS and FS for H_2O dissociation on Mg(0001) surface.

	E_{ad}	G_{ad}	$d_{O\text{-}Mg}$	$d_{H\text{-}Mg}$	$d_{O\text{-}H}$
H_2O*	-0.46	-0.08	2.178		0.981, 0.982
OH*+H*	-2.08	-1.62	2.109, 2.093, 2.081	1.994, 1.992, 2.009	0.978
O*+2H*	-2.78	-2.31	1.981, 1.951, 1.954, 2.111	1.973, 1.988, 2.029, 1.973, 2.025, 1.988	
$O+H_2$	-2.21	-1.86	1.960, 1.965, 1.963, 2.099		

Table S6 Computed dissociation barriers and energies (E_a and E_r, eV), dissociation Gibbs free energy barriers and Gibbs free energies (G_a and G_r, eV), bond distances ($d_{Mg\text{-}X}$, Å), the breaking O-H bond distance ($d_{H\text{-}O}$, Å) and forming H-H bond distance ($d_{H\text{-}H}$, Å) in the transition state for H_2O dissociation on Mg(0001) surface.

	reaction	E_a (G_a)	E_r (G_r)	$d_{O\text{-}Mg}$	$d_{H\text{-}Mg}$	$d_{H\text{-}O}$	$d_{H\text{-}H}$
TS1	$H_2O*\rightarrow OH*+H*$	0.40 (0.45)	-1.62 (-1.54)	2.124, 2.127	2.241, 2.119	1.300	
TS2	$OH*+H*\rightarrow O*+2H*$	1.19 (1.19)	-0.70 (-0.69)	2.025, 2.004, 1.967	1.927, 2.173, 2.311	1.413	
TS3	$O*+2H*\rightarrow O*+H_2*$	1.12 (1.11)	0.57 (0.45)	1.964, 1.963, 1.963	2.095, 1.944, 1.948, 2.089		1.210
TS4	$OH*+H*\rightarrow O*+H_2*$	0.77 (0.76)	-0.13 (-0.24)	2.011, 2.009, 2.009	2.282, 2.277, 2.023,	1.239	1.035

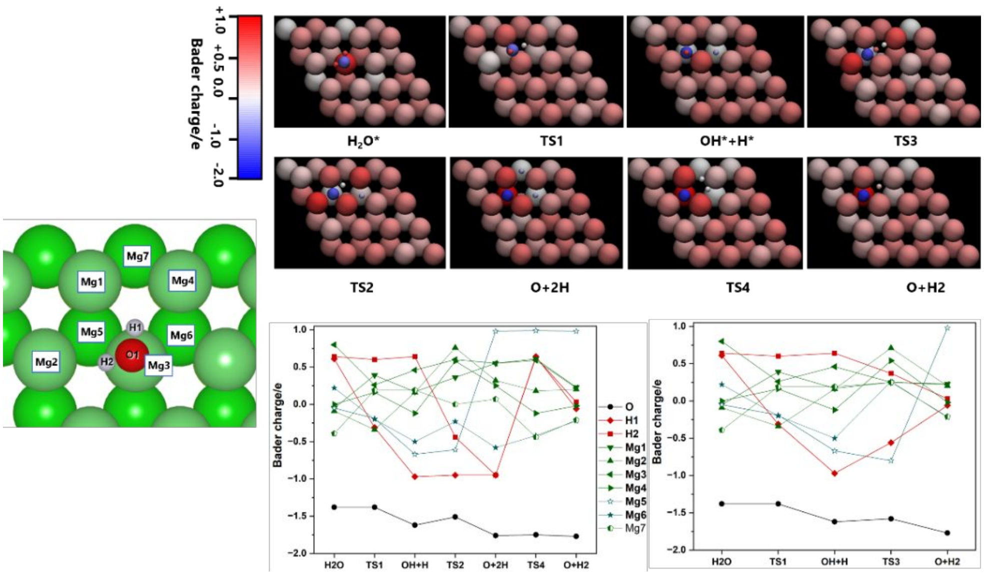

Fig. S6 Bader charge evolution during H_2O dissociation on Magnesium surface. Atoms which appear to take part in the reaction were labeled, and their Bader charge was plotted with respect to the reaction coordinate.

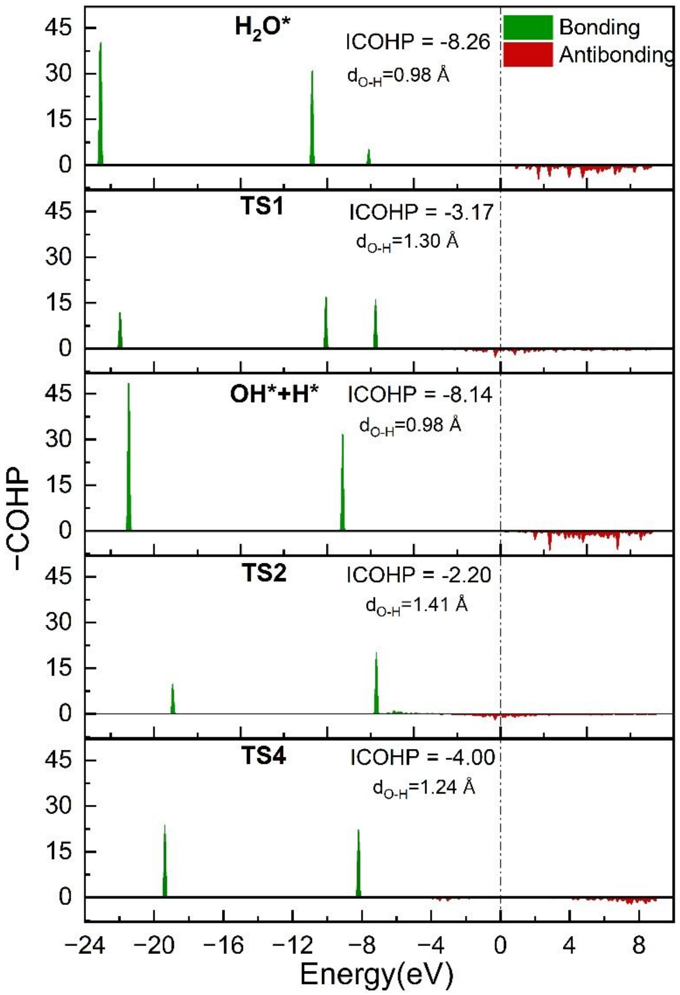

Fig.S7 COHP curves of O-H bond during H_2O dissociation on pure Mg(0001) surface.

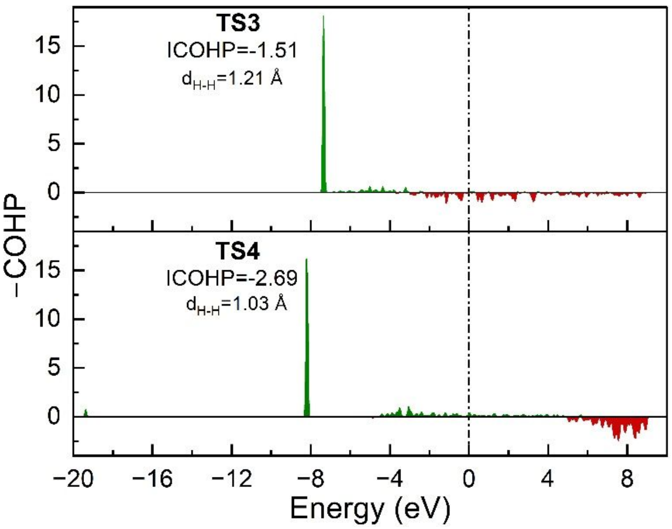

Fig.S8 COHP curves of H-H bond during H_2O dissociation on pure Mg(0001) surface.

Table S7 Adsorption energy (E_{ads}, eV), adsorption Gibbs free energy (G_{ads}, eV) and shortest distances ($d_{H\text{-}Mg}$, Å) of the IS and FS for $2H_2O$ dissociation on Mg(0001) surface.

	E_{ads}	G_{ads}	$d_{O\text{-}Mg}$	$d_{H\text{-}Mg}$	$d_{H\text{-}O}$
H_2O+H_2O	-1.00	-0.18	2.337, 2.158		0.982, 0.989, 0.989, 0.978
$H_2O+OH+H$	-2.60	-1.75	2.222, 2.069, 2.094, 2.075	1.967,1.985,1.999	0.979, 0.984
H_2O+O+H_2	-2.82	-2.01	2.165, 1.963, 1.964, 1.961		0.979, 0.987
2OH+2H	-4.28	-3.37	2.102, 2.111, 2.062, 2.065, 2.121, 2.092	2.032, 1.983, 2.003, 1.984, 1.997, 2.019	0.978, 0.978
$2OH+H_2$	-3.78	-2.94	2.108, 2.099, 2.076, 2.080, 2.095, 2.108		0.978, 0.978

Table S8. Computed dissociation barriers and energies (E_a and E_r, eV), dissociation Gibbs free energy barriers and Gibbs free energies (G_a and G_r, eV), bond distances ($d_{Mg\text{-}X}$, Å), the breaking O-H bond distance ($d_{O\text{-}H}$, Å) and forming H-H bond distance ($d_{H\text{-}H}$, Å) in the transition state for $2H_2O$ dissociation on Mg(0001) surface.

	Reaction path	E_a (G_a)	E_r (G_r)	$d_{O\text{-}Mg}$	$d_{H\text{-}Mg}$	$d_{H\text{-}O}$	$d_{H\text{-}H}$
TS5	$2H_2O \rightarrow H_2O + OH + H$	0.28 (0.32)	-1.60 (-1.57)	2.070, 2.103	2.236, 2.181, 2.286	1.357	-
TS6	$H_2O + OH + H \rightarrow H_2O + O + H_2$	0.82 (0.84)	-0.22 (-0.26)	2.173, 1.998, 2.009, 2.013	2.267, 2.275, 2.196,	1.238	1.045
TS7	$H_2O + OH + H \rightarrow 2OH + 2H$	0.40 (0.43)	-1.68 (-1.62)	2.117, 2.134, 2.064, 2.186, 2.124,	1.918, 1.976, 2.006, 2.234, 2.201,	1.268	-
TS8	$H_2O + OH + H \rightarrow 2OH + H_2$	0.31 (0.34)	-1.17 (-1.19)	2.097, 2.107, 2.062, 2.156, 2.279	2.341, 1.984, 2.442, 2.453	1.126	1.188
TS9	$2OH + 2H \rightarrow OH + O + 3H$	1.31 (1.31)	-0.69 (-0.69)	2.026, 2.008, 1.960, 2.071, 2.131, 2.034	1.899, 2.462	1.432	-
TS10	$2OH + 2H \rightarrow 2OH + H_2$	1.25 (1.23)	0.50 (0.43)	2.105, 2.090, 2.069, 2.077, 2.068, 2.116	2.098, 1.985, 1.898, 2.268	-	1.146
TS11	$2OH + 2H \rightarrow OH + O + H + H_2$	0.77 (0.76)	-0.16 (-0.19)	2.004, 1.996, 2.012, 2.063, 2.111, 2.077	2.132, 2.471, 2.175	1.225	1.055

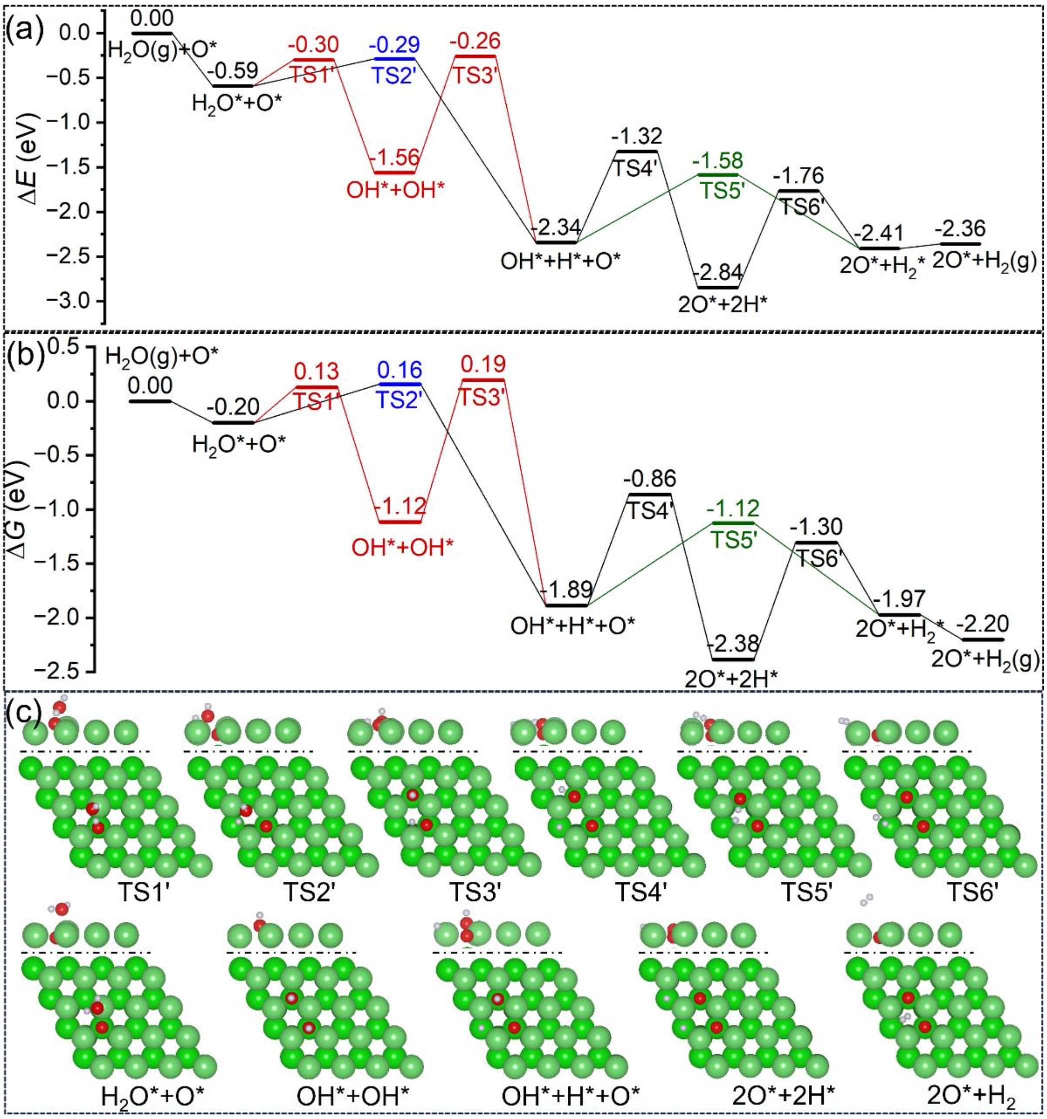

Fig. S9 Potential energy surfaces (a); Change of Gibbs free energy (b) and corresponding adsorption configuration (c) (top: side view of the first layer, bottom: top view; Mg/green; O/red; H/white) for H_2O dissociative adsorption on O pre covered Mg(0001) surface(* for surface species, g for gas phase species)

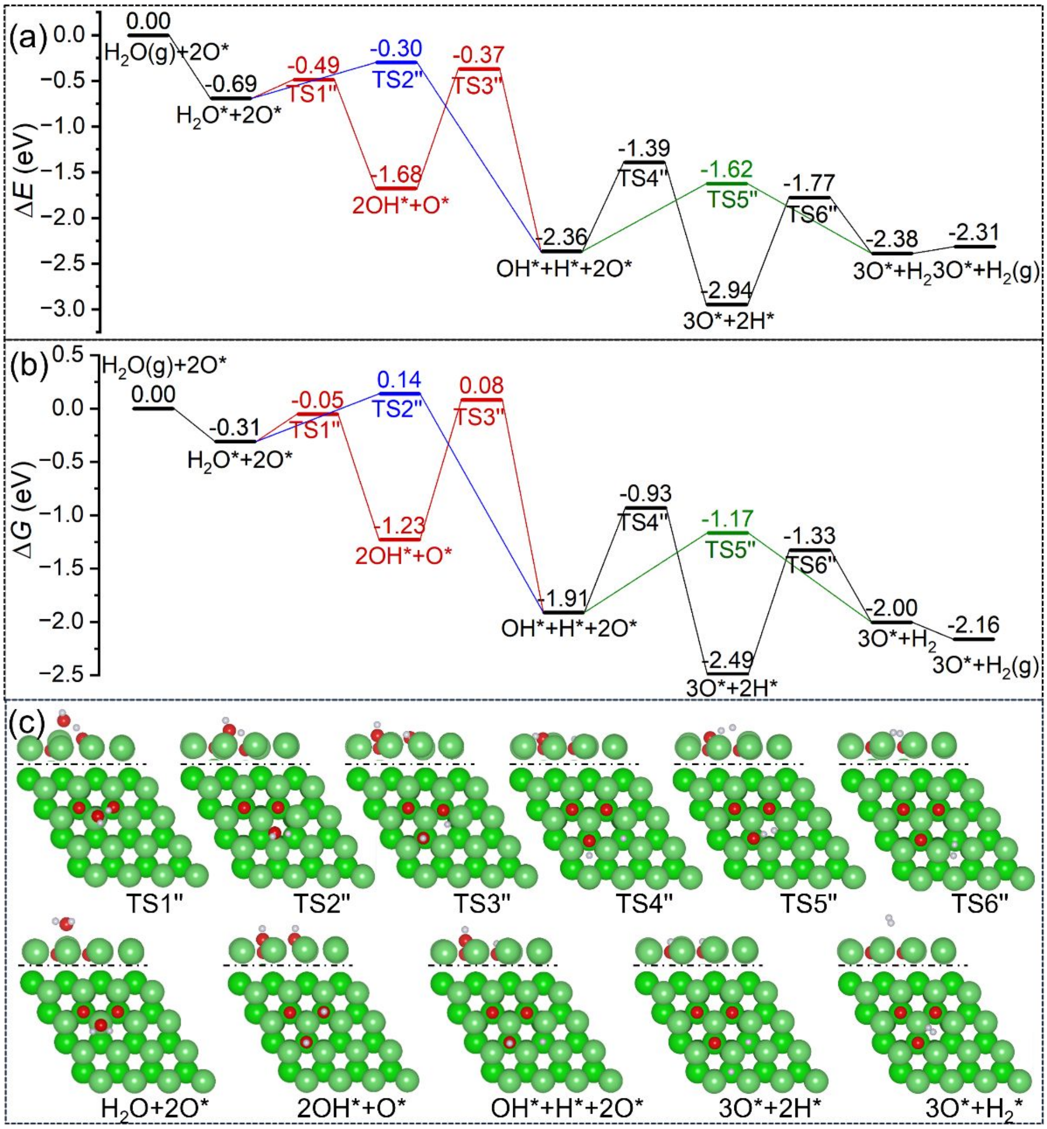

Fig. S10 Potential energy surfaces (a); Change of Gibbs free energy (b) and corresponding adsorption configuration (c) (top: side view of the first layer, bottom: top view; Mg/green; O/red; H/white) for H_2O dissociative adsorption on 2×O pre covered Mg(0001) surface(* for surface species, g for gas phase species)

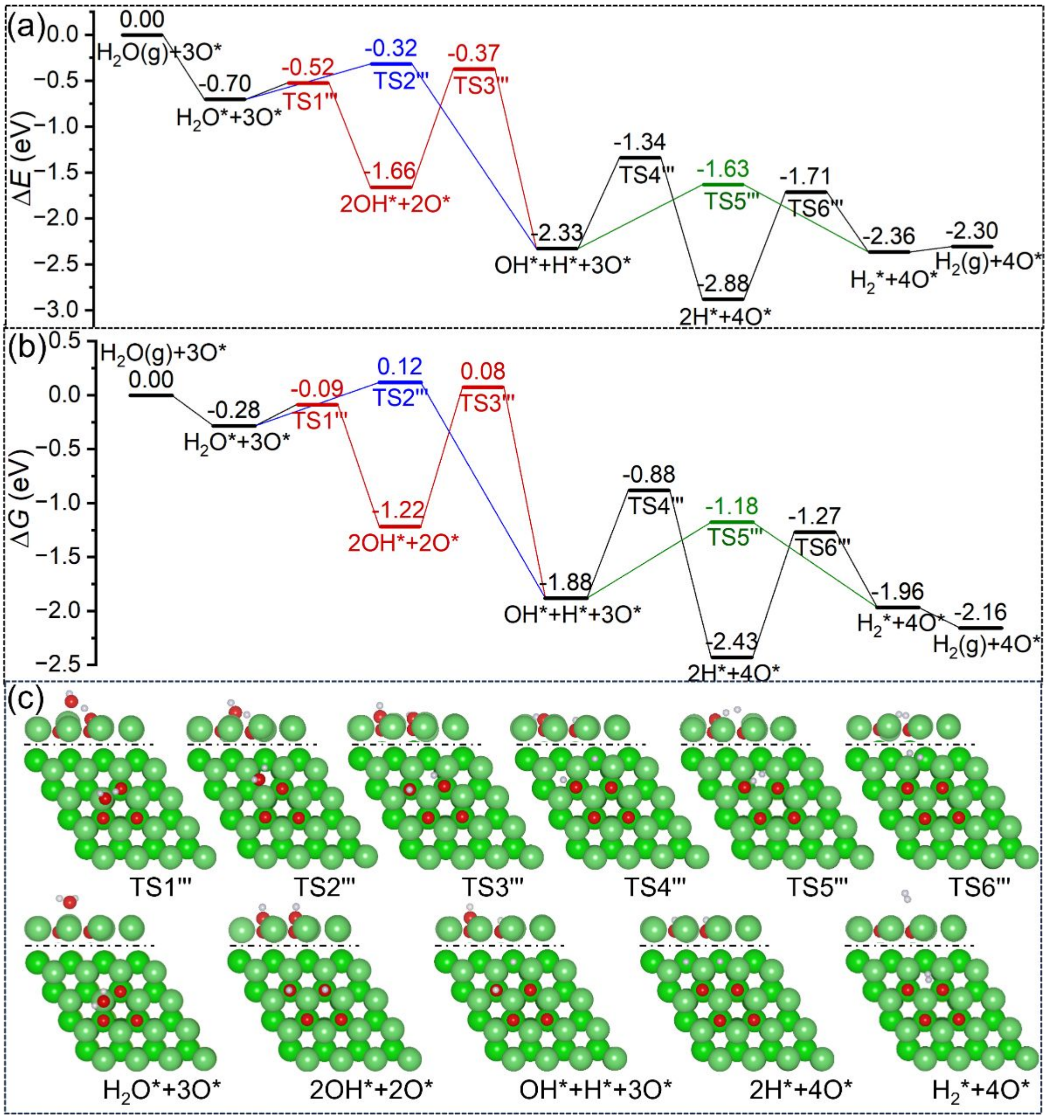

Fig. S11 Potential energy surfaces (a); Change of Gibbs free energy (b) and corresponding adsorption configuration (c) (top: side view of the first layer, bottom: top view; Mg/green; O/red; H/white) for H_2O dissociative adsorption on 3×O pre covered Mg(0001) surface (* for surface species, g for gas phase species)

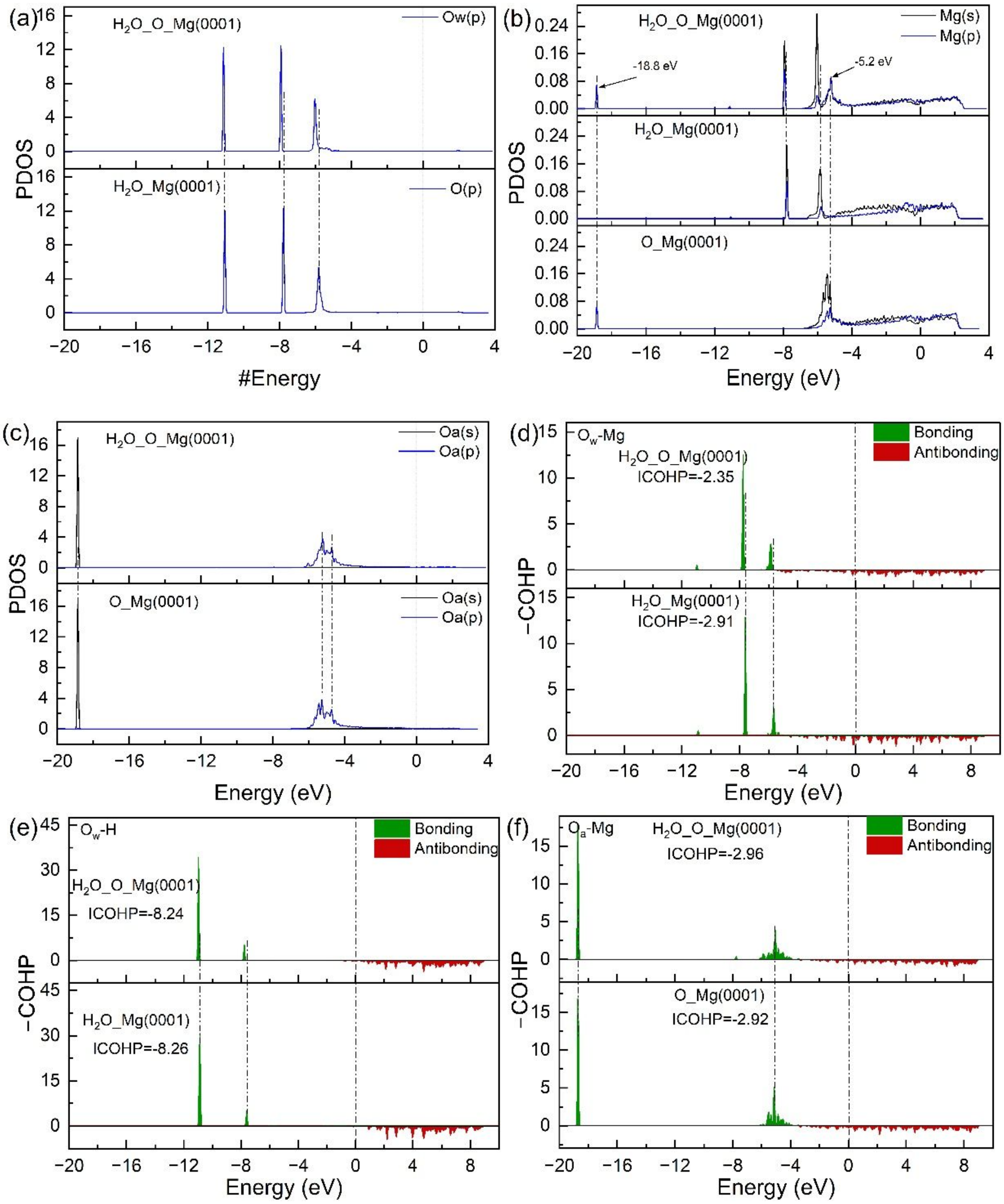

Fig.S12 (a) Comparisons of PDOS plots of the Mg atom of water adsorption on pure Mg(0001) surface (H_2O_ Mg(0001)) and on O pre-covered Mg0001 surface (H_2O_O_ Mg(0001)), clean O pre-covered surface(O_Mg(0001)); (b) Comparisons of PDOS plots of the O_w atom of water adsorption on pure Mg(0001) surface (H_2O_Mg0001) and on O pre-covered Mg0001 surface (H_2O_O_Mg0001); (c) Comparisons of PDOS plots of the O_a atom of water adsorption on O pre-covered Mg0001 surface (H_2O_O_Mg0001) and the clean O pre-covered surface (O_Mg(0001); (d) Comparisons of COHP curves of O_w-Mg bond of water adsorption on pure Mg(0001) surface (H_2O_Mg0001) and O pre-covered Mg(0001) surface (H_2O_O_ Mg(0001)); (e) Comparisons of COHP curves of O_w-H bond of water adsorption on pure Mg(0001) surface (H_2O_Mg(0001)) and O pre-covered Mg(0001) surface (H_2O_O_Mg(0001)); (f) Comparisons of COHP curves of O_a-Mg bond of water adsorption on O pre-covered Mg(0001) surface (H_2O_O_Mg(0001)) and the clean O pre-covered Mg(0001) surface (O_Mg(0001))

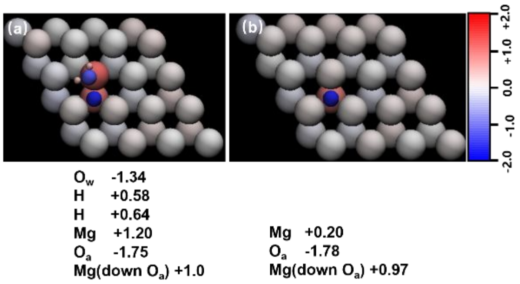

Fig. S13 Bader charge distributions of (a) H_2O adsorbed on O pre-covered Mg(0001) surface; (b) O pre-covered Mg(0001) surface （The text under the picture indicated the Bader charge of the atoms）

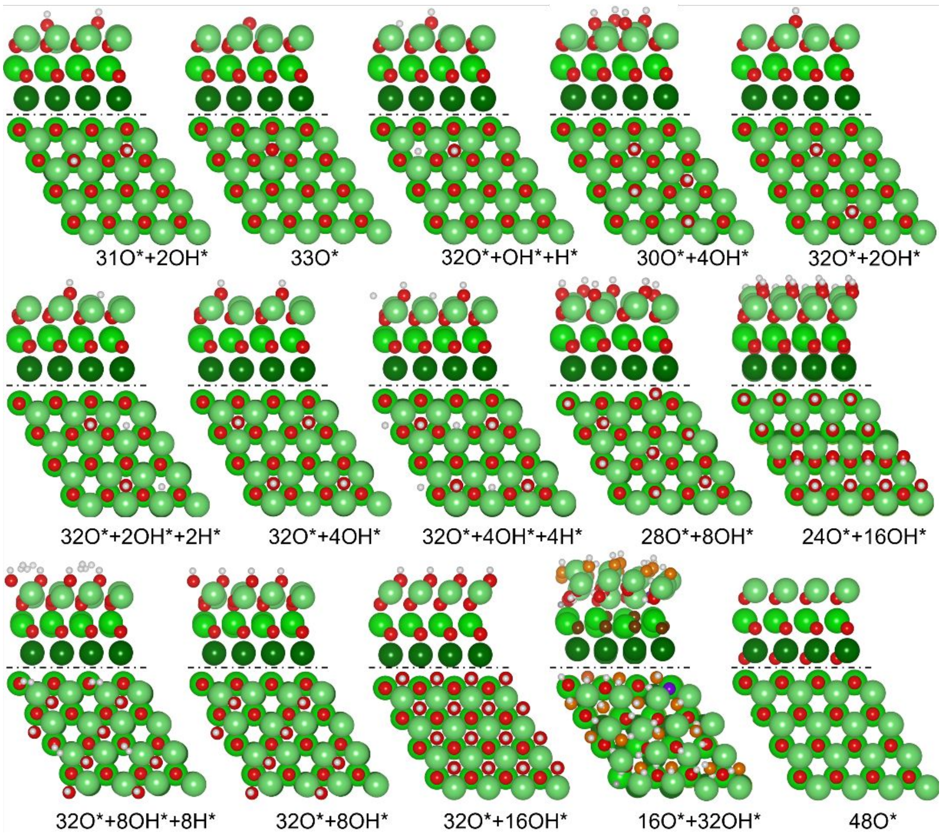

Fig.S14 Adsorption configuration (top: side view of the top three layers, bottom: top view; Mg/green; O/red; H/white) for water dissociative adsorption on 32O pre-adsorbed Mg(0001) surface (* for surface species, g for gas phase species)
